# *Panax quinquefolius* saponin inhibits vascular smooth muscle cell calcification via activation of nuclear factor-erythroid 2-related factor 2

**DOI:** 10.1186/s12906-023-03961-6

**Published:** 2023-04-21

**Authors:** Xiaoting Lu, Xue Liu, Ershun Liang, Ruixue Yang, Yan Liu, Xiaoqiong Liu, Fangfang Yan, Yifan Xing

**Affiliations:** 1 The Key Laboratory of Cardiovascular Remodeling and Function Research, Chinese Ministry of Education, Chinese National Health Commission and Chinese Academy of Medical Sciences, The State and Shandong Province Joint Key Laboratory of Translational Cardiovascular Medicine, Department of Cardiology, Qilu Hospital of Shandong University, Shandong 250012 Jinan, China; 2 Department of Traditional Chinese Medicine, Qilu Hospital of Shandong University, Jinan, 250012 Shandong China; 3 Department of Medical Insurance, Qilu Hospital of Shandong University, Jinan, 250012 Shandong China

**Keywords:** *Panax quinquefolius* saponin, Nuclear factor-erythroid 2-related factor 2, Oxidative stress, Vascular smooth muscle cell, Calcification

## Abstract

**Background:**

*Panax quinquefolius* saponin (PQS) is the main active component of *Panax quinquefolius*. Emerging evidence suggests that PQS exerts beneficial effects against cardiovascular diseases. However, the role and mechanism of PQS in vascular calcification are not unclear. The present study investigated the effects of PQS on the calcification of vascular smooth muscle cell (VSMCs).

**Methods:**

The present study used calcification medium containing 3 mM inorganic phosphate (Pi) to induce rat VSMCs calcification. We investigated the effects of PQS on VSMCs calcification using alizarin red staining and alkaline phosphatase (ALP) activity assays. The intracellular reactive oxygen species (ROS) levels and the transcriptional activity of nuclear factor-erythroid 2-related factor 2 (Nrf2) were determined. The mRNA and protein expression levels of Nrf2, the antioxidant gene heme oxygenase-1 (HO-1), osteogenic markers, including runt-related transcription factor 2 (Runx2) and bone morphogenetic protein 2 (BMP2), and Kelch-like ECH-associated protein 1 (Keap1) were also measured.

**Results:**

Treatment with Pi significantly increased intracellular calcium deposition and ALP activity, which were suppressed by PQS in a concentration-dependent manner. During VSMCs calcification, PQS inhibited the mRNA and protein expression of Runx2 and BMP2. PQS treatment reduced intracellular ROS production and significantly upregulated Nrf2 transcriptional activity and the expression of Nrf2 and its target antioxidant gene HO-1. PQS suppressed the Pi-induced protein expression of Keap1, which is an endogenous inhibitor of Nrf2. Keap1 siRNA treatment induced Nrf2 expression and downregulated Runx2 expression in the presence of Pi and PQS.

**Conclusion:**

Taken together, these findings suggest that PQS could effectively inhibit VSMCs calcification by ameliorating oxidative stress and regulating osteogenic genes via the promotion of Nrf2 expression.

**Supplementary Information:**

The online version contains supplementary material available at 10.1186/s12906-023-03961-6.

## Background

Vascular calcification is a major cause of increasing mortality in patients with diabetes, atherosclerosis, hypertension, and chronic kidney disease (CKD) [1, 2]. The prevalence of vascular calcification increases with age, and it is approximately 60% for individuals > 70 years of age [3, 4]. Cumulative evidence suggests that vascular calcification is a strictly regulated process that is similar to bone formation [5, 6]. Its characteristics include the deposition of calcium phosphate in arteries, the phenotypic transformation of vascular smooth muscle cell (VSMCs) into osteoblast-like cells, and the expression of bone-related proteins [7, 8]. Increased stiffness and decreased elastic recoil in the aortic wall lead to reduced coronary perfusion and concentric ventricular hypertrophy [9]. However, the underlying mechanisms of vascular calcification have not been established.

Oxidative stress is a critical regulator of many age-related diseases, including vascular calcification [10]. The production of reactive oxygen species (ROS) and the oxidative modification of various biomolecules induce the transformation of VSMCs from the contractile type to the osteogenic type, which is accompanied by calcification [7]. For example, hydrogen peroxide (H_2_O_2_) is a classic oxidant stressor that promotes vascular cell calcification by increasing the expression of the osteogenic transcription factor runt-related transcription factor 2 (Runx2) [11]. The reduction in ROS by 4-hydroxy-2,2,6,6,-tetramethyl piperidinoxyl (tempol), a membrane-permeable antioxidant, blocks VSMCs differentiation into osteoblast-like cells [12]. The transcription factor nuclear factor-erythroid 2-related factor 2 (Nrf2), which belongs to the Cap'n'collar/basic region leucine zipper (CNC-bZIP) transcription factor family, plays a negative role in VSMCs calcification by inhibiting oxidative stress and interacting with the osteogenic transcription factor Runx2 [13–15].

*Panax quinquefolius* saponin (PQS) is the main active component of *Panax quinquefolius*. Emerging evidence suggests that PQS exerts pleiotropic beneficial effects on cardiovascular diseases and diabetes, and its protective effects may be mostly attributable to its antioxidant effects [16–18]. One previous study found that PQS protected the myocardium against myocardial infarction by reducing oxidative stress injury and suppressing excessive endoplasmic reticulum stress [19]. *Panax quinquefolius* also inhibited oxidative stress-induced cardiomyocyte death by activating the Nrf2 signaling pathway [20]. However, whether PQS prevents VSMCs calcification is not known. Therefore, the present study tested the hypothesis that PQS blocked VSMCs calcification via activation of Nrf2.

## Methods

### Materials and reagents

Standardized PQS was supplied by Jilin Yisheng Pharmaceutical Co., Ltd. (Jilin Province, China). Dulbecco's modified Eagle’s medium (DMEM) and fetal bovine serum (FBS) were purchased from Gibco (Grand Island, NY, USA). TRIzol reagent and a Prime Script RT Reagent Kit were purchased from Invitrogen (Carlsbad, CA, USA). Alizarin red S was obtained from Sigma (cat. no. A5533, St. Louis, MO, USA). A Dual-Luciferase Reporter Assay System was purchased from Promega (cat. no. E1910, WI, USA). Rabbit polyclonal anti-Nrf2 (cat. no. sc-13032, H300; 1:500), anti-β-actin (cat. no. sc-47778; 1:1000) and goat anti-rabbit IgG (cat. no. sc-2004; 1:5000) were purchased from Santa Cruz (Dallas, TX, USA). Primary antibodies against proteins including Runx2 (cat. no. ab23981; 1:1000), bone morphogenetic protein 2 (BMP2) (cat. no. ab14933, H300; 1:1000), SM22α (cat. no. ab14106; 1:1000) and heme oxygenase-1 (HO-1) (cat. no. ab13243; 1:2000) were purchased from Abcam (Cambridge, MA, USA).

### Cell isolation and identification

Aortic smooth muscle cells were separated from the thoracic aorta of 6-week-old male Sprague–Dawley (SD) rats. Each rat was anesthetized via abdominal injection of a 1% sodium pentobarbital solution (10 ml/kg), and the thoracic aorta was removed and minced into small pieces (1–2 mm^2^). The pieces were transferred to digestive enzymes mixing 0.2% trypsin and 0.1% collagenase I solution at 37 °C for 20 min with shaking. The process of digestion was terminated by adding 5 ml 10% fetal bovine serum (FBS). The cells attached to the dish were collected and cultured in DMEM supplemented with 10% FBS and antibiotics in a 95% humidified-air incubator at 37 °C with 5% CO_2_. VSMCs were identified by positive staining of α-smooth muscle actin and used for the experiments between passages 5–8.

### Calcification induction and cell treatment

VSMCs were seeded in culture dishes and maintained in DMEM with 10% FBS. After confluency, cells were incubated in calcification medium containing 3 mM Pi for 6 days to induce calcification. The first day of culture in the calcification medium was defined as day 0. PQS was added to the culture medium at different concentrations during the calcification period. The treatment medium was changed every 2 days. Cells cultured in DMEM supplemented with 10% FBS without Pi and PQS were used as the blank group.

### Cell viability assay

Cell viability was determined using an MTS assay kit (Promega, WI, USA) according to the manufacturer’s instructions. VSMCs were seeded 96-well plates and incubated for 24 h. The culture medium was removed and replaced with DMEM with or without PQS at different concentrations (0, 25, 50, 100 and 200 μg/ml) for 24 h. Then, the medium was removed, followed by incubation with 100 μl of fresh 10% FBS medium and 20 μl of MTS cell viability reagents. After incubation at 37 °C for 1 h, the absorbance at 490 nm was measured using a multiwell spectrophotometer. All values were normalized to the control group.

### Alizarin red staining

Alizarin red staining was used for the quantitative detection of calcium deposition in VSMCs. Briefly, after treatment for 6 days, the cells were washed with phosphate-buffered saline (PBS) 3 times, fixed in 4% neutral formalin for 30 min and stained with 1% alizarin red (pH 4.2) for 5 min at room temperature. The cells were washed with PBS 3 times to remove nonspecific staining and the cells were photographed under light microscopy.

### Alkaline phosphatase (ALP) activity assay

To quantify intracellular calcium deposition, ALP activity was determined using an Alkaline Phosphatase Assay Kit (cat. no. P0321M, Beyotime Biotechnology, Hangzhou, China) in accordance with the manufacturer's instructions. Briefly, lysis buffer (20 mM Tris, pH 7.5; 150 mM NaCl; 1% Triton X-100) was added to the cells, which were incubated for 30 min at 4 °C and centrifuged at 111.8 rcf for 10 min. The supernatants were transferred to a 96-well plate and incubated with para-nitrophenyl phosphate (pNPP) for 10 min at 37 °C. The stop solution was added to the reaction mixture, and ALP activity was determined using a microplate reader at an absorbance wavelength of 405 nm (BioTek Synergy, VT, USA). The amount of total protein in the lysate was quantified using a bicinchoninic acid (BCA) assay kit (Sigma, MO, USA). The ALP activity was normalized to the protein content. The specific activity to produce 1 nmol of p-nitrophenol was defined as one unit, and the values of ALP activities are expressed as units/mg protein.

### Reactive oxygen species (ROS) assay

Intracellular ROS levels were measured using a ROS Assay Kit (cat. no. S0033M, Beyotime Biotechnology, Hangzhou, China) according to the instructions. VSMCs were incubated with Pi (3 mM) in the presence or absence of PQS for 48 h and then treated with 10 μM DCFH-DA at 37 °C for 20 min. The cells were washed with DMEM 3 times, and the fluorescence intensity of the cell lysates was measured using a fluorescence microplate reader at an excitation wavelength of 488 nm and an emission wavelength of 525 nm. The results are expressed as a percentage of the control value.

### Nrf2 transcriptional reporter assay

An Nrf2 transcriptional reporter assay was performed using a Dual-Luciferase Reporter Assay System. Briefly, VSMCs were transfected with ARE-luc (firefly luciferase) plasmids containing the Nrf2 reporter gene with Lipofectamine 2000. After 48 h of transfection, the luciferase activity was determined using a Dual-Glo Luciferase Assay System. The pRL-TK-luc (Renilla luciferase) plasmids were used as an internal control, and Nrf2 transcriptional activity was expressed by normalizing the luciferase values to the empty vector control values.

### Small interfering RNAs transfection

The small interfering RNA (siRNA) for Nrf2, Keap1 and the negative control were purchased from GenePharma (Shanghai, China). Cells were transiently transfected with siRNAs using the Lipofectamine RNAiMAX (Invitrogen) according to the manufacturer's instructions. After 48 h, cells were cultured in calcification medium with or without PQS and utilized for the further experiments.

### Quantitative real-time PCR (RT-PCR)

According to the manufacturer's instructions, total RNA from cultured cells was isolated with TRIzol reagent and used to synthesize cDNA with a reverse transcription kit. A SYBR Premix Ex Taq Kit (Takara, Dalian, China) was used to perform RT-PCR according to the manufacturer's protocol. The cycling conditions used were as follows: predenaturation at 95 °C for 30 s followed by 40 cycles of 95 °C for 5 s and 60 °C for 34 s. The amplification of mRNA was analyzed using the 2^−ΔΔCt^ method. The expression levels of target genes were normalized to the mRNA levels of the internal reference β-actin. The primer sequences of the genes are listed in Table 1.

**Table 1:** Primer sequences for real-time PCR

Genes	Sequence (5’-3’)	Size (bp)	Gene ID
**Nrf2**	GCTATTTTCCATTCCCGA	109	NM_031789
	ATTGCTGTCCATCTCTGTCAG		
**HO-1**	AGAGTTTCTTCGCCAGAGG	127	NM_012580
	GAGTGTGAGGACCCATCG		
**Runx2**	TCGGAAAGGGACGAGAG	101	NM_001278483
	TTCAAACGCATACCTGCAT		
**BMP2**	AAGCCAGGTGTCTCCAAG	209	NM_017178
	AAGTCCACATACAAAGGGTG		
**SM22α**	CTGTAATGGCTTTGGGCAGT	97	NM_031549
	CTCTTATGCTCCTGGGCTTTC		
**β-actin**	ATGGTGGTATGGGTCAGAAGG	264	NM_031144
	TGGCTGGGGTGTTGAAGGTC		

*Nrf2* Nuclear factor-erythroid 2-related factor 2, *HO-1* Heme oxygenase-1, *Runx2* Runt-related transcription factor 2, *BMP2* Bone morphogenetic protein 2, *SM22α* Smooth muscle 22 alpha

### Western blot analysis

Total cell protein was extracted in RIPA lysis buffer containing protease inhibitors, and the concentration of protein was quantified using a BCA assay kit according to the manufacturer's protocol. Equal amounts of protein were separated by 10% SDS-PAGE and transferred to PVDF membranes, which were blocked in 5% milk for 2 h and incubated with specific primary antibodies overnight at 4 °C. After washing 3 times in TBST, the membranes were incubated with the appropriate secondary antibodies at room temperature for 1 h. The protein bands were visualized with enhanced chemiluminescence reagent.

### Statistical analysis

Unless otherwise indicated, data were obtained from at least 3 separate experiments performed in triplicate. Measurement data are presented as the mean ± standard deviation (SD). Analyses were performed using SPSS 17.0 statistical software (IBM Corporation, Armonk, NY, USA). Statistical significance was determined using one-way ANOVA followed by Tukey’s t test. A value of *p* < 0.05 indicated statistical significance.

## Results

### PQS prevents high phosphate-induced vascular smooth muscle cell calcification

**Fig. 1:**
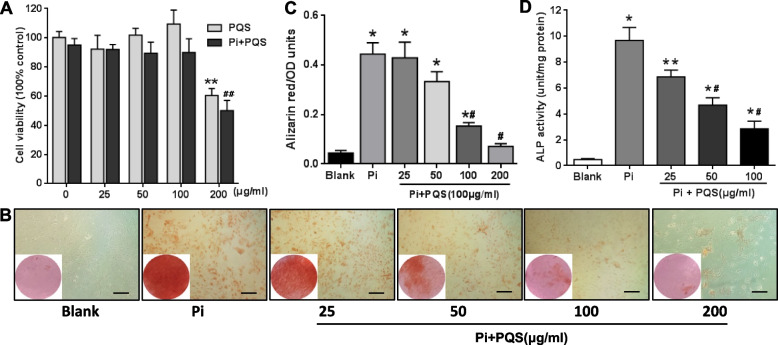
PQS prevents high phosphate-induced vascular smooth muscle cell calcification. VSMCs were treated with increasing concentrations of PQS (0, 25, 50, and 100 μg/ml) in the presence of calcification medium containing 3 mM Pi for 6 days. **A** Cell viability was measured using MTS assay following treatment of VSMCs with increasing concentrations of PQS for 24 h. **B** Calcium deposition was assessed using alizarin red staining. Scale bar: 100 μm. **C** Quantification of alizarin red staining. **D** Quantification of calcium deposition using ALP activity assay. The values are presented as the mean ± SD (*n* = 3). ^*^p < 0.05 or ^**^p < 0.01 *vs.* blank, and ^#^*p* < 0.05 or ^##^*p* < 0.01 *vs.* Pi control or PQS control

We first investigated the toxicity of PQS and Pi in VSMCs. Cells were treated with PQS at various concentrations (0, 25, 50, 100 and 200 μg/ml) for 24 h. After treatment, cell viability was determined by MTS assay. As shown in Fig. 1A, in the PQS alone groups, 200 μg/ml PQS decreased cell viability by 39.82% (**p < 0.01). VSMCs exposed to 200 μg/ml PQS and 3 mM Pi medium exhibited a 47.35% (^##^*p* < 0.01) decrease compared with that of Pi treatment cells. Cell viability was not affected by 25, 50 and 100 μg/ml PQS with or without Pi treatment. The results of the MTS assay demonstrated that Pi and PQS alone or in combination did not impair cell viability up to a concentration of 100 μg/ml.

To examine the effect of PQS on calcification in VSMCs, cells were incubated in calcification medium with increasing concentrations of PQS for 6 days. Alizarin red staining revealed no calcium deposition in the absence of Pi, but incubation with 3 mM Pi significantly increased the calcium content (Fig. 1B). According to the quantitative analysis, the calcium levels in the PQS groups were reduced by 3.33%, 24.94% and 65.35% (^#^*p* < 0.05) in 25, 50 and 100 μg/ml PQS groups, respectively, compared with the Pi group (Fig. 1C). Although calcium deposition was inhibited by 200 μg/ml PQS, cell toxicity was also increased, and cell calcification was further reduced.

Similar to the alizarin red staining results, PQS significantly decreased ALP activity in a concentration-dependent manner (Fig. 1D). Compared to the Pi control, 25, 50 and 100 μg/ml PQS decreased secreted ALP activity by 29.25%, 51.71% (^#^*p* < 0.05) and 70.47% (^#^*p* < 0.05), respectively, and the most effective concentration was 100 μg/ml. Therefore, PQS concentrations between 25 μg/ml and 100 μg/ml were used to treat cells in subsequent experiments.

### PQS inhibits high phosphate-induced expression of bone formation markers

**Fig. 2:**
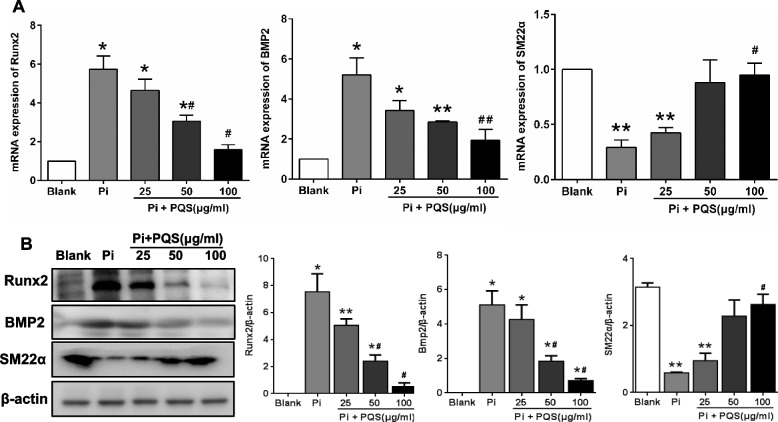
PQS inhibits high phosphate-induced expression of calcification markers. VSMCs were treated with various concentrations of PQS (0, 25, 50, and 100 μg/ml) in the presence of calcification medium containing 3 mM Pi for 6 days. The expression of osteogenic marker genes Runx2 and BMP2, and VSMCs marker gene SM22α was determined using RT-PCR (**A**) and Western blot analysis (**B**). The relative values of protein expression were normalized to β-actin. ^*^p < 0.05 or ^**^p < 0.01 *vs.* blank, and ^#^*p* < 0.05 or ^##^*p* < 0.01 *vs.* Pi control

To investigate the effect of PQS on VSMCs differentiation, the genes associated with calcification were examined using RT‒PCR and Western blot analysis. Following intervention with increasing concentrations of PQS for 6 days, the mRNA expression of the bone formation markers Runx2 and BMP2 was significantly inhibited. Compared to the Pi control group, Runx2 and BMP2 mRNA levels were decreased by 72.46% (^#^*p* < 0.05) and 62.82% (^##^*p* < 0.01), respectively, in the 100 μg/ml PQS group. Conversely, the mRNA expression of SM22α, a specific marker of smooth muscle cell, was markedly increased by 0.46, 2.04 and 2.28-fold (^#^*p* < 0.05) in the PQS treatment groups. PQS (100 μg/ml) maintained SM22α expression up to the levels of the blank group (Fig. 2A). Similar to the mRNA expression changes, 100 μg/ml PQS decreased the protein expression levels of Runx2 and BMP2 by 92.98% (^#^*p* < 0.05) and 86.21% (^#^*p* < 0.05), respectively, and the levels of SM22α were increased by 3.48-fold compared with the Pi control group (^#^*p* < 0.05) (Fig. 2B).

**Fig. 3:**
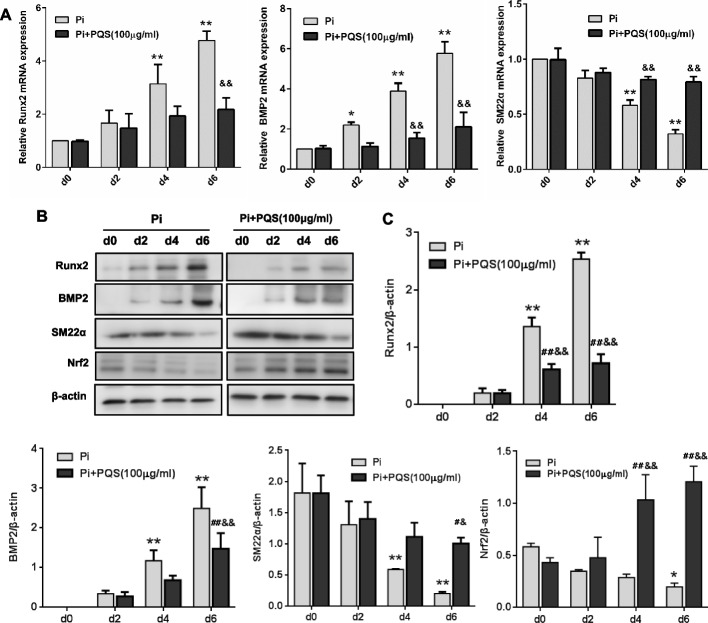
PQS inhibits high phosphate-induced expression of calcification markers and Nrf2 during calcification period. VSMCs were induced to calcify with or without 100 μg/ml PQS and harvested at different times (d0, d2, d4 and d6). **A** The mRNA expression of osteogenic marker genes during calcification was determined using RT-PCR. **B** The protein expression of osteogenic marker genes and Nrf2 during calcification was determined using Western blot analysis. The relative values of protein expression were normalized to β-actin. ^*^p < 0.05 or ^**^p < 0.01 *vs.* Pi control on d0, ^#^*p* < 0.05 or ^##^*p* < 0.01 *vs.* Pi + PQS group on d0, and ^&^*p* < 0.05 or ^&&^*p* < 0.01 *vs.* Pi control on d0, d2, d4 and d6*.* The values are presented as the means ± SD (*n* = 3)

We further examined the time course responses of Runx2, BMP2 and SM22α during calcification. VSMCs were treated in high-phosphate medium with PQS (100 μg/ml) and harvested at the indicated stages (d0, d2, d4, and d6). In contrast to the Pi control group at the same time point, the PQS treatment group exhibited inhibition of the mRNA expression of Runx2 and BMP2 by 54.45% (^&&^*p* < 0.01) and 63.52% (^&&^*p* < 0.01), respectively, and upregulation of SM22α by 1.46-fold (^&&^*p* < 0.01) on d6 (Fig. 3A). The protein expression levels of Runx2, BMP2, and SM22α were consistent with the mRNA expression levels (Fig. 3B and C).

Meanwhile, to identify the antioxidant signals involved in calcification, we investigated the effects of Pi and PQS on the activation of Nrf2, a critical regulator of antioxidant responses, during different calcification periods. Western blot analyses and quantification of the bands showed that 3 mM Pi decreased the protein level of Nrf2 by 66.07% (^*^p < 0.05), whereas PQS further increased Nrf2 protein expression by 1.8-fold (^##^*p* < 0.01) after inducing calcification for 6 days (Fig. 3B and C). These findings suggested that PQS attenuated Pi-induced calcification by reducing calcification marker expression in VSMCs.

### PQS attenuates high phosphate-induced vascular smooth muscle cell calcification by activating Nrf2

**Fig. 4:**
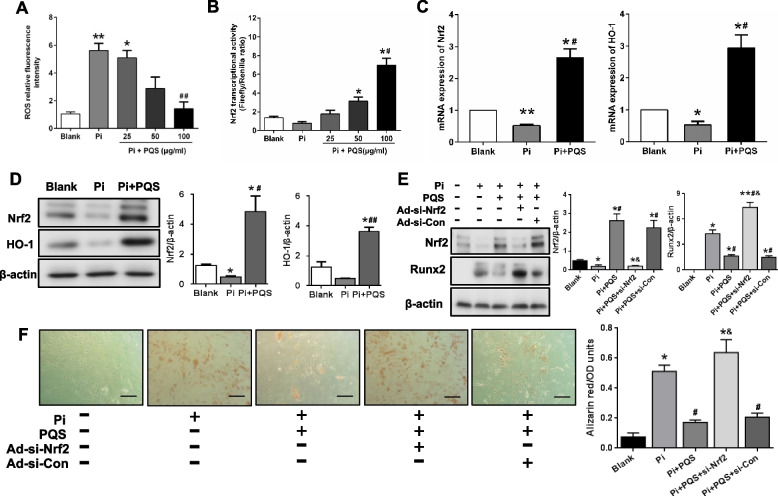
PQS attenuates high phosphate-induced vascular smooth muscle cell calcification by activating Nrf2. **A** VSMCs were incubated with 3 mM P in the presence or absence of PQS for 48 h, and intracellular ROS levels were determined as the mean fluorescence intensity. ^*^p < 0.05 or ^**^p < 0.01 *vs.* blank, and ^##^*p* < 0.01 *vs.* Pi control*.* **B** VSMCs were transfected with the Nrf2 transcription reporter gene ARE-luc and the internal control gene pRL-TK-luc then treated with various concentrations of PQS for 48 h. Nrf2 transcriptional activity was assessed using dual luciferase assay. ^*^p < 0.05 *vs.* blank, and ^#^*p* < 0.05 *vs.* Pi control*.* **C** and **D** Cells were treated with calcification medium containing 3 mM Pi for 6 days. The expression of Nrf2 and HO-1 was determined using RT-PCR and Western blot analysis. ^*^p < 0.05 *vs.* blank, and ^#^*p* < 0.05 or ^##^*p* < 0.01 *vs.* Pi control*.* (E and F) VSMCs were transfected with Nrf2-siRNA or control siRNA for 48 h, and further incubated with or without PQS in calcification medium for 6 days. The expression of Runx2, SM22α and Nrf2 was determined using Western blot analysis. Calcium deposition was visualized by alizarin red staining. Scale bar: 200 μm. The values are presented as the means ± SD (*n* = 3). ^*^p < 0.05 or ^**^p < 0.01 *vs.* blank, ^#^*p* < 0.05 *vs.* Pi control, and ^&^*p* < 0.05 *vs.* Pi + PQS

To investigate whether oxidative stress is involved in vascular calcification, we observed the effect of PQS on ROS production. As shown in Fig. 4A, ROS production was significantly increased by 4.37-fold (^**^p < 0.01) in the presence of high phosphate, but this effect was profoundly reduced by 74.75% (^##^*p* < 0.01)after 100 μg/ml PQS treatment. These results suggest that PQS ameliorates oxidative stress during VSMCs calcification.

Nrf2 plays an important role in inhibiting ROS generation. Next, we examined whether PQS treatment alone activated Nrf2 in VSMCs. Compared to the blank group, Nrf2 transcriptional activity was inhibited in the Pi-treated group. After VSMCs were incubated with various concentrations of PQS for 48 h, Nrf2 transcriptional activity was activated in a dose-dependent manner, and maximal activation was achieved at a dose of 100 µg/ml (Fig. 4B). Therefore, we examined the effects of PQS on the expression of Nrf2 and its target antioxidant gene HO-1 using PQS at 100 µg/ml. As shown in Fig. 4C, Pi strongly inhibited the mRNA expression of Nrf2 by 48.01% (^*^p < 0.05) and HO-1 by 47.30% (^*^p < 0.05), which were increased by 4.10- (^#^*p* < 0.05) and 4.58 -fold (^#^*p* < 0.05), respectively, in the PQS treatment group compared to the Pi group. These protein levels are comparable with those of mRNA expression. Compared with the Pi group, PQS increased Nrf2 and HO-1 protein levels by 8.81- (^#^*p* < 0.05) and 6.51-fold (^##^*p* < 0.01), respectively (Fig. 4D). These results suggested that PQS treatment reduced ROS production and activated Nrf2 in VSMCs.

To further study the role of Nrf2 in high phosphate-induced vascular calcification, we inhibited Nrf2 expression by transfecting cells with a small interfering RNA against Nrf2 (siNrf2). As shown in Fig. 4E, western blotting demonstrated that the protein level of Runx2 was substantially increased by 0.73- (^#^*p* < 0.05) and by 3.47 -fold (^&^*p* < 0.05) in response to 3 mM Pi in the presence of siNrf2 treatment compared with that of the Pi control and PQS groups. The inhibitory effect of PQS on calcification was decreased after Nrf2 gene silencing. Alizarin red staining (Fig. 4F) revealed that siNrf2-treated cells showed significantly more mineral deposition after Pi stimulation for 6 days than scramble-transfected cells, which further suggested that VSMCs calcification was aggravated in the absence of Nrf2. Briefly, our results demonstrated that the inhibition of calcification by PQS was mediated via the activation of Nrf2.

### PQS regulates the Keap1/Nrf2 pathway in high phosphate-induced vascular smooth muscle cell calcification

**Fig. 5:**
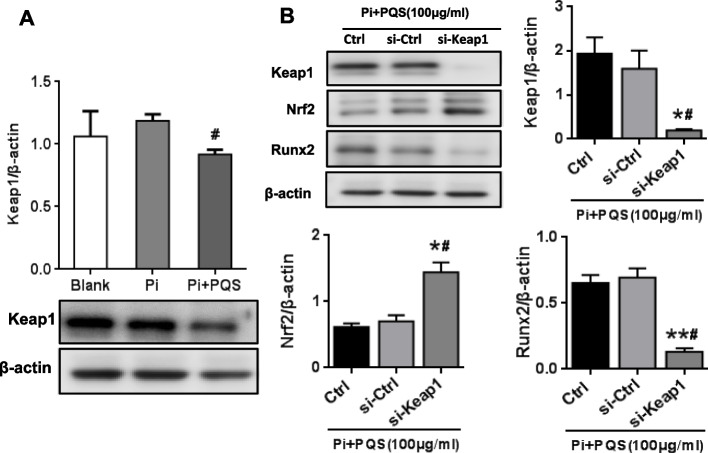
PQS regulates the Keap1/Nrf2 pathway during high phosphate-induced vascular smooth muscle cell calcification. **A** VSMCs were treated with calcification medium containing 3 mM Pi for 6 days. The protein expression of Keap1 was determined using Western blot analysis. ^#^*p* < 0.05 *vs.* Pi control*.* **B** VSMCs were transfected with Keap1 siRNA or control siRNA and further incubated with PQS in calcification medium for 6 days. The protein expression of Keap1, Nrf2 and the osteogenic marker Runx2 was determined using Western blot analysis. The values are presented as the means ± SD (*n* = 3). ^*^p < 0.05 or ^**^p < 0.01 *vs.* Ctrl, ^#^*p* < 0.05 *vs.* si-Ctrl

To gain further insight into the mechanisms by which PQS inhibits VSMCs calcification, we investigated whether PQS activates Nrf2 expression by regulating the Keap1-Nrf2 system. Compared to the Pi group, PQS suppressed Pi-induced Keap1 protein expression by 22.83% (*p < 0.05), while Pi showed no inhibitory effect on Keap1 (Fig. 5A). Transfection of Keap1 siRNA in the presence of Pi and PQS increased the protein expression of Nrf2 by 1.07-fold (^#^*p* < 0.05) and significantly inhibited Runx2 expression by 81.08% (^#^*p* < 0.05) (Fig. 5B). These results indicated that the inhibitory effect of PQS on VSMCs calcification was mediated by regulation of the Keap1/Nrf2 pathway, although other pathways could not be excluded.

## Discussion

In the present study, we investigated the effects of PQS on VSMCs calcification in vitro and the underlying mechanisms. We demonstrated that PQS effectively prevented the Pi-induced mineralization process and inhibited the expression of osteogenic marker genes by reducing oxidative stress, activating the expression of Nrf2 and decreasing the protein level of Keap1. Blockade of Nrf2 partially abolished the beneficial effects of PQS on calcification. Taken together, our findings suggest that PQS inhibits VSMCs calcification and is associated with attenuation of oxidative stress via the Nrf2/Keap1 pathway.

Inorganic phosphate is essential for many cellular processes, and elevated serum phosphorus plays an important role in the progression of vascular calcification [21]. In this study, high concentrations of Pi were used to induce VSMCs calcification, which is morphologically similar to that observed in the calcified human aortic media and heart valves [22, 23]. The underlying molecular mechanisms might be related to VSMCs phenotypic transition mediated by phosphate cotranspor Pit-1 and apoptosis mediated by a secreted prote Growth arrest-specific gene 6 (Gas6) [24, 25]. Oxidative stress is caused by ROS generation that exceeds local antioxidant capacity. Accumulating studies have demonstrated that oxidative stress is involved in many of the molecular events of vascular calcification [26–28]. For example, selenite prevents vascular calcification by inhibiting oxidative stress-induced activation of the phosphatidylinositol 3-kinase (PI3K)/AKT and extracellular regulated kinase (ERK) signaling pathways and endoplasmic reticulum stress, which leads to decreased osteoblastic differentiation and apoptosis of VSMCs [26]. The inhibition of dynamin-related protein 1 (DRP1), which is a key regulator of mitochondrial fission, attenuates oxidative stress-mediated mitochondrial dysfunction, matrix mineralization, and cytoskeletal rearrangement, which reduces cardiovascular calcification [27]. Quercetin attenuates VSMCs apoptosis and calcification by inhibiting oxidative stress and decreasing mitochondrial fission [28]. In the presence of high Pi concentrations, ROS homeostasis is imbalanced, and overproduction of ROS leads to a cell apoptosis cascade and eventually different vascular pathologies, including VSMCs osteochondrogenic transdifferentiation, inflammation, and extracellular matrix remodeling [29, 30]. We found that PQS treatment reduced the production of ROS and vascular calcification caused by Pi in a dose-dependent manner. This result suggests that PQS protects VSMCs against oxidative injury during the process of calcification.

Nrf2, a master transcription factor, suppresses oxidative stress by controlling the expression of numerous antioxidant and detoxification genes, including HO-1, glutathione (GSH), and thioredoxin (TXN) [31]. Under normal conditions, Nrf2 binds to Keap1, which is an important regulator of the ubiquitylation and degradation of Nrf2. Under perturbed conditions such as oxidative stress, Keap1 is inactivated, which results in Nrf2 dissociation and nuclear translocation [32]. In summary, Nrf2 decreases intracellular ROS levels through its antioxidant activity and plays a fundamental role in maintaining cellular redox homeostasis. According to the results of previous studies, activation of Nrf2 may be beneficial to attenuate VSMCs calcification [33–35]. Alpha-lipoic acid, an Nrf2 activator, attenuates calcification in VSMCs and mice by restoring mitochondrial function and the intracellular redox state via its antioxidant potential [36]. Dimethyl fumarate (DMF) stimulates Nrf2 activity to attenuate VSMCs calcification by inhibiting osteogenic genes [15]. Our data revealed that the expression levels of Nrf2 and HO-1 were significantly increased after 6 days of stimulation by PQS, which suggested that Nrf2 is critically involved in the development of VSMCs calcification. Nrf2 knockdown by siRNA increased calcium deposition, reversed the expression of Runx2 and suppressed the inhibitory effect of PQS on calcification in VSMCs. The finding that PQS inhibits VSMCs calcification by activating Nrf2 and suppressing oxidative stress establishes a direct mechanistic link between PQS-mediated vascular protective effects and antioxidative activity. Sheng et al. discovered that the activation of the Nrf2-ARE signaling pathway may inhibit vascular calcification via the suppression of Runx2 and BMP2 [37]. Our results are in accordance with other findings that the expression of osteogenic marker genes, including Runx2 and BMP2, is highly correlated with Nrf2 levels during VSMCs calcification. Keap1 plays a critical role in the inhibition of Nrf2 activity. PQS inhibited Keap1 expression, which might be the mechanism of elevated Nrf2 expression. However, Nrf2 activity is also regulated via Keap1-independent or other pathways, including the βTrCP-CUL1 complex, the WD40 repeat-containing protein 23 (WDR23) complex, and P62 transcription [38–40]. Future studies should focus on delineating other signaling pathways, especially those closely correlated with cell apoptosis and inflammation in ginseng.

*Panax quinquefolius*, also called American ginseng in Asia, is native to the United States and Canada [41]. It is an important herb that has been widely used to prevent and treat diseases for hundreds of years [42]. PQS exerts protective effects against cardiovascular diseases by attenuating oxidative stress injury, protecting ischemic and reperfused myocardial tissue, increasing energy storage in the myocardium, reducing myocardial apoptosis, and improving ventricular reconstruction [20, 43, 44]. Rb1, Rg1, and Rb2 are the main ginsenosides in ginseng that exhibit a remarkable antioxidant effect via the activation of the Nrf2 pathway [45–48]. In addition, Sun et al. reported that ginsenoside Rb3 protects cardiomyocytes against hypoxia/reoxygenation-induced oxidative stress by activating the antioxidation signaling pathway of PERK/Nrf2/HMOX1 [49]. PQS treatment significantly attenuated the elevation of malonyldialdehyde (MDA) and superoxide dismutase (SOD) induced by intermittent high glucose in human umbilical vein endothelial cell (HUVECs) through the phosphatidylinositol 3-kinase kinase (PI3K)/Akt/GSK-3β pathway [50]. All these studies demonstrate that the Nrf2 pathway is considered a key molecular mechanism by which PQS attenuates oxidative stress injury in many organs and tissues. Unfortunately, so far there is no data about PQS for the treatment of vascular calcification in human. However, PQS are major bioactive components of Xinyue capsule which is a patented Chinese herbal medicine and is used as adjunct to conventional therapy on cardiovascular diseases for over ten years in China [51]. Previous study demonstrated that in patients with stable coronary artery diseases (CAD) after percutaneous coronary intervention (PCI) within the preceding 3 to 12 months, Xinyue capsule (100 mg PQS, three times a day) reduced the incidence of primary composite endpoint in addition to conventional treatment [52]. Our study demonstrated that PQS inhibited high phosphate-induced VSMCs calcification in vitro, further studies are needed to determine whether PQS prevents vascular calcification in vivo and to provide theoretical evidence for PQS as a potential therapy in patients with vascular calcification.

## Conclusion

In summary, the results of the present study revealed that activation of Nrf2 signaling was likely a crucial pathway for the PQS-mediated inhibition of VSMCs calcification. PQS effectively inhibited VSMCs calcification by ameliorating oxidative stress and regulating osteogenic genes via the promotion of Nrf2 expression. Therefore, PQS against oxidative stress may offer a greater therapeutic benefit for vascular calcification with an improved side-effect profile.

## Supplementary Information

**Additional file 1.**

**Additional file 2.**

**Additional file 3.**

**Additional file 4.**

**Additional file 5.**

**Additional file 6.**

## Data Availability

All data and materials used to support the findings of this study are included in this article.

## Abbreviations

PQS: *Panax quinquefolius* Saponin
VSMCs: Vascular smooth muscle cell
Pi: Inorganic phosphate
ALP: Alkaline phosphatase
ROS: Reactive oxygen species
Nrf2: Nuclear factor-erythroid 2-related factor 2
HO-1: Heme oxygenase-1
Runx2: Runt-related transcription factor 2
BMP2: Bone morphogenetic protein 2
Keap1: Kelch-like ECH-associated protein 1
CKD: Chronic kidney disease
H_2_O_2_: Hydrogen peroxide
DMEM: Dulbecco's modified Eagle medium
SD: Sprague–Dawley
PBS: Phosphate-buffered saline
pNPP: Para-nitrophenyl phosphate
BCA: Bicinchoninic acid
SD: Standard deviation
GSH: Glutathione
TXN: Thioredoxin
DMF: Dimethyl fumarate
MDA: Malonyldialdehyde
SOD: Superoxide dismutase
HUVECs: Human umbilical vein endothelial cell
PI3K: Phosphatidylinositol 3-kinase kinase
CAD: Coronary artery diseases
