# Supplementary material for: Panax quinquefolius saponin inhibits vascular smooth muscle cell calcification via activation of nuclear factor-erythroid 2-related factor 2
Source: BMC Complement Med Ther. 2023 Apr 21;23:129. doi: 10.1186/s12906-023-03961-6 (PMC10120105; doi:10.1186/s12906-023-03961-6)
Supplement: Supplementary file 6 — Additional file 6. [file 12906_2023_3961_MOESM6_ESM.pptx]

## Slide 1
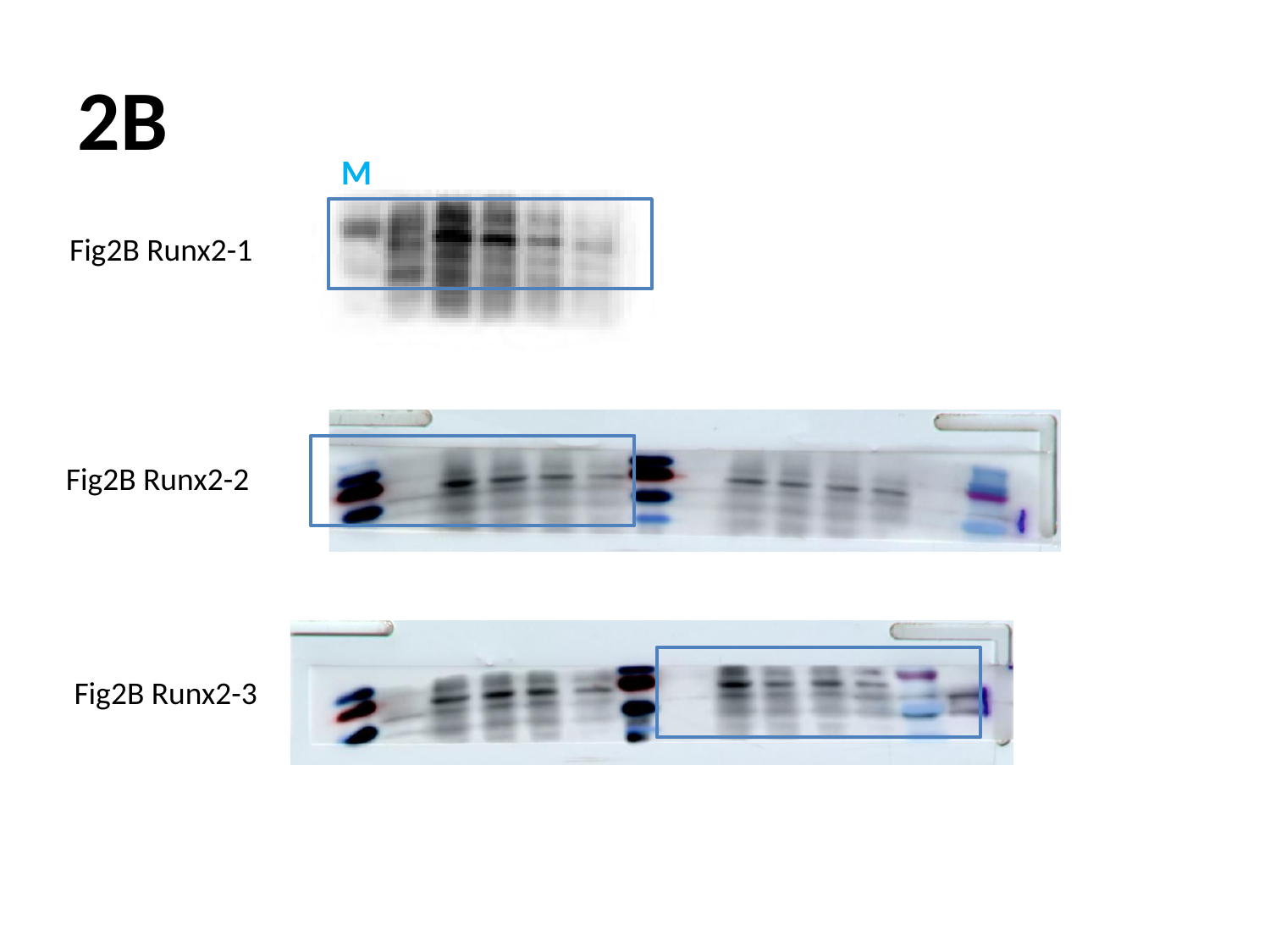

2B
M
Fig2B Runx2-1
Fig2B Runx2-2
Fig2B Runx2-3

## Slide 2
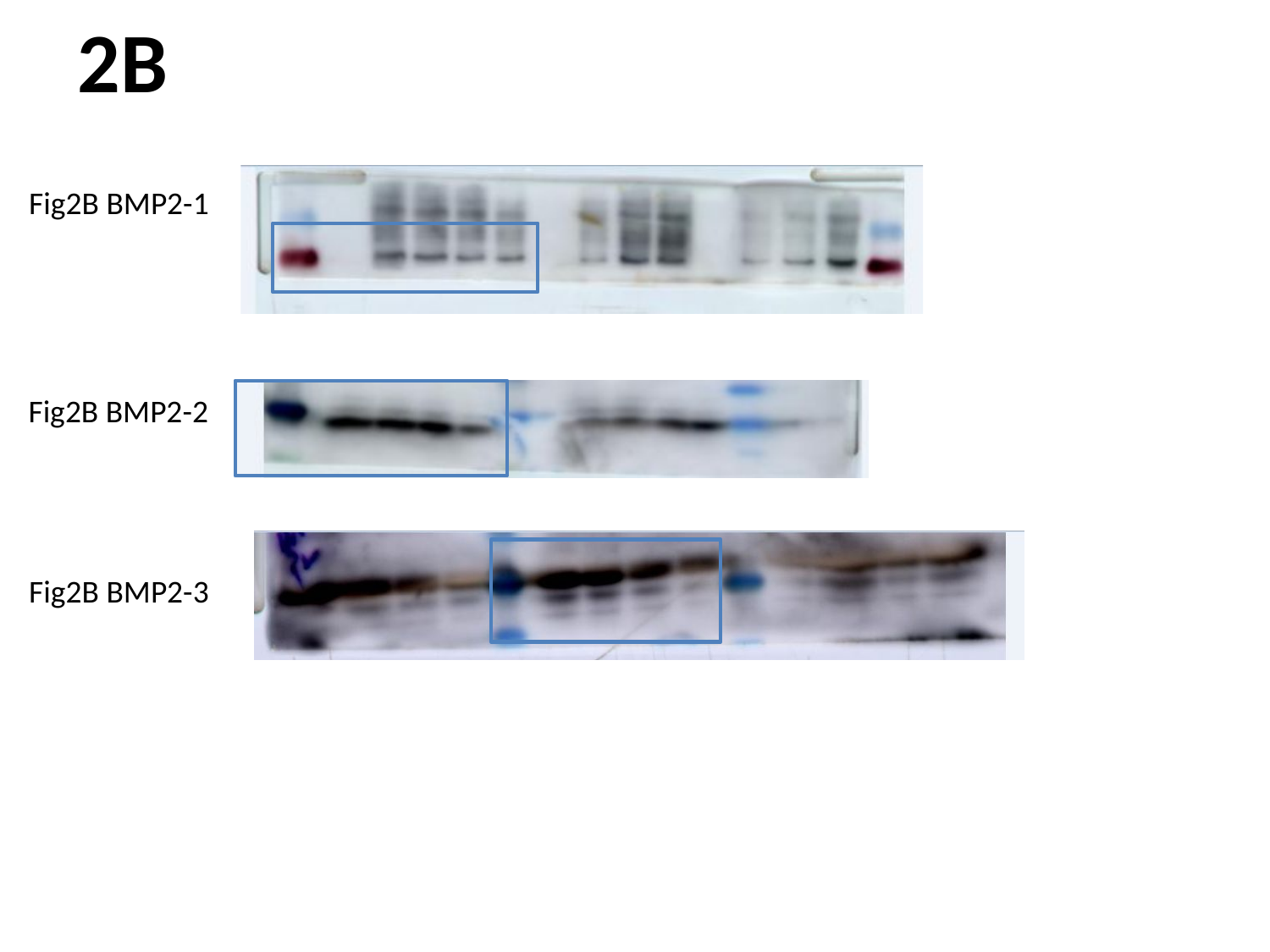

2B
Fig2B BMP2-1
Fig2B BMP2-2
Fig2B BMP2-3

## Slide 3
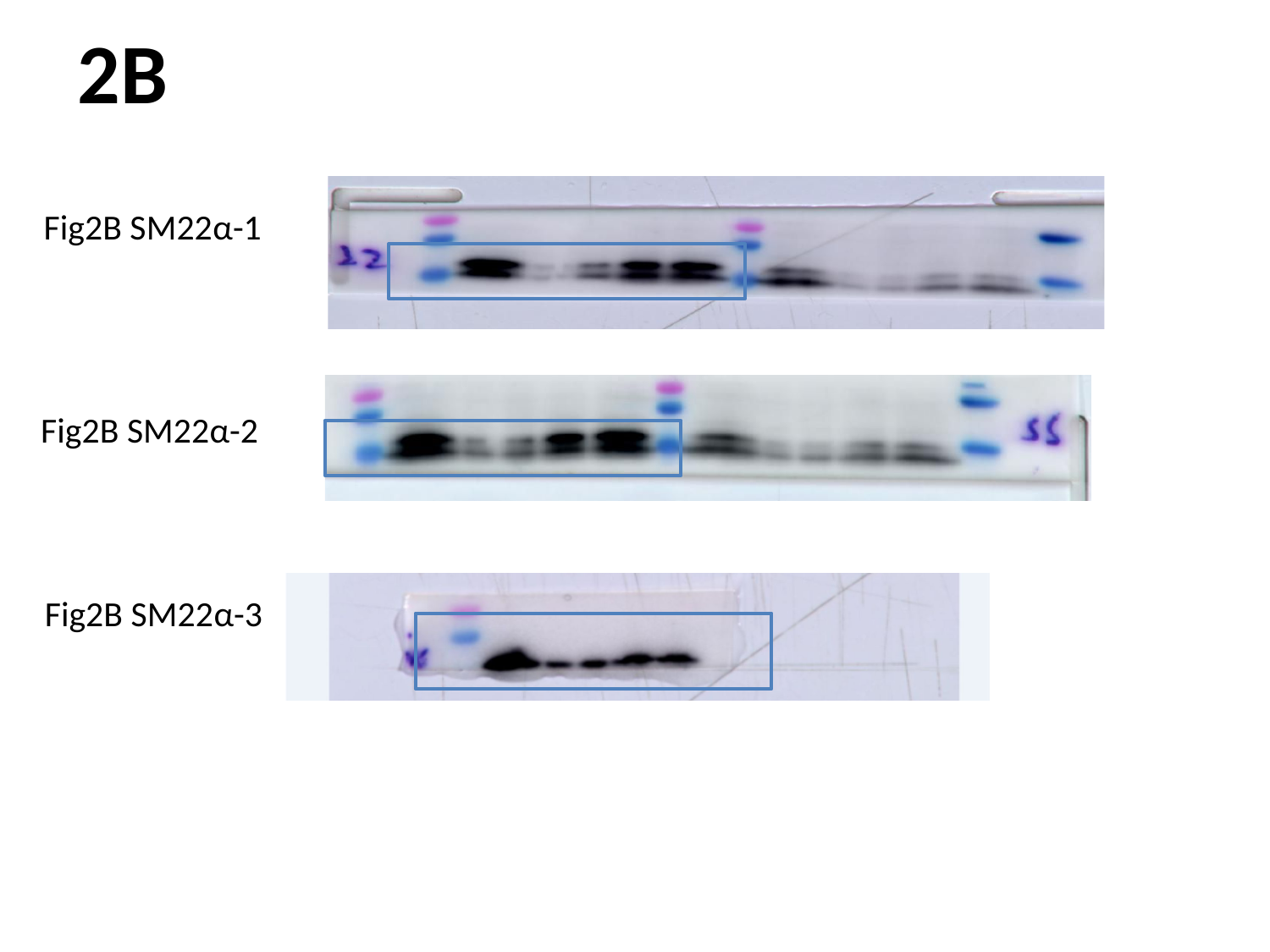

2B
Fig2B SM22α-1
Fig2B SM22α-2
Fig2B SM22α-3

## Slide 4
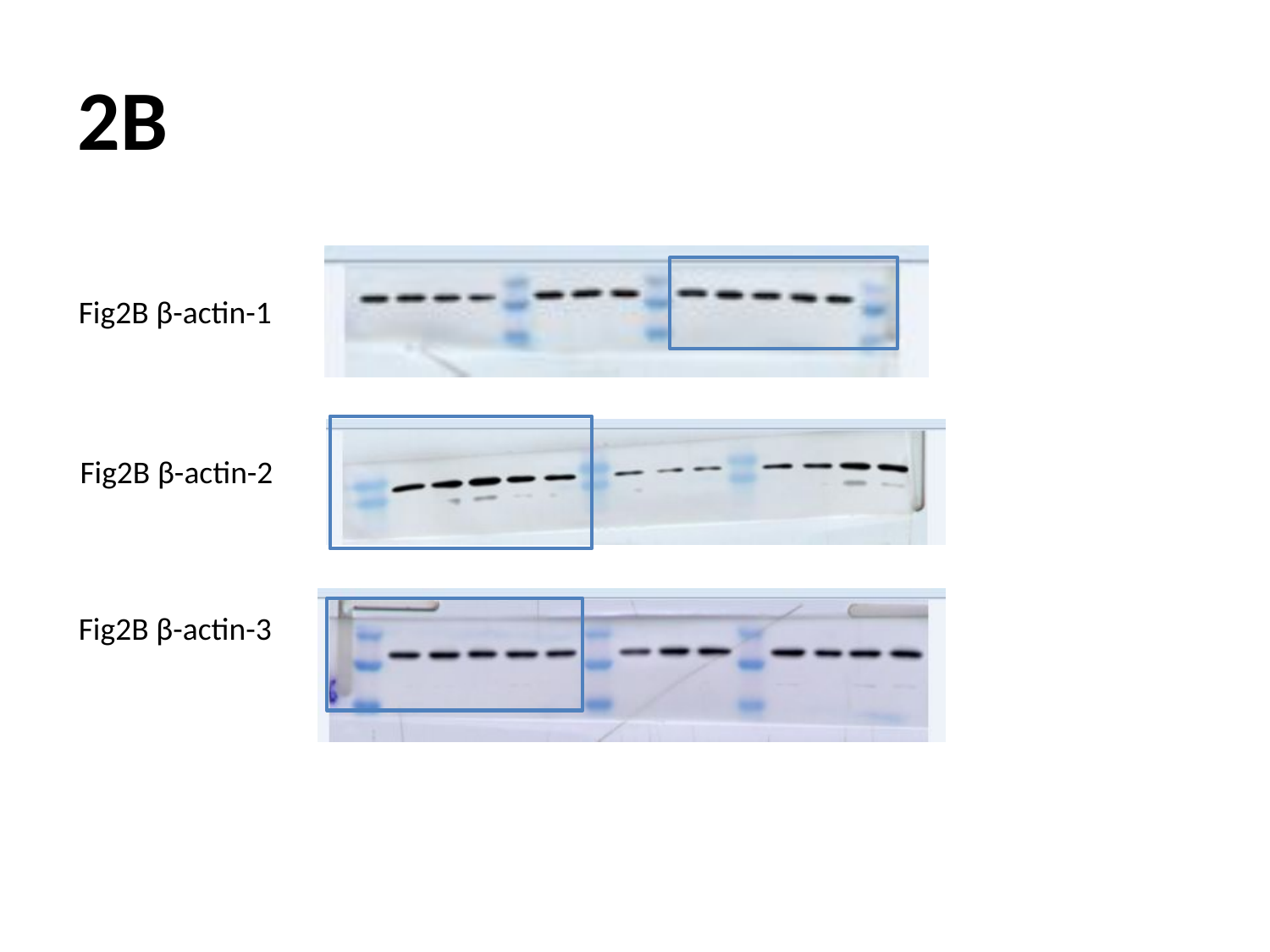

2B
Fig2B β-actin-1
Fig2B β-actin-2
Fig2B β-actin-3

## Slide 5
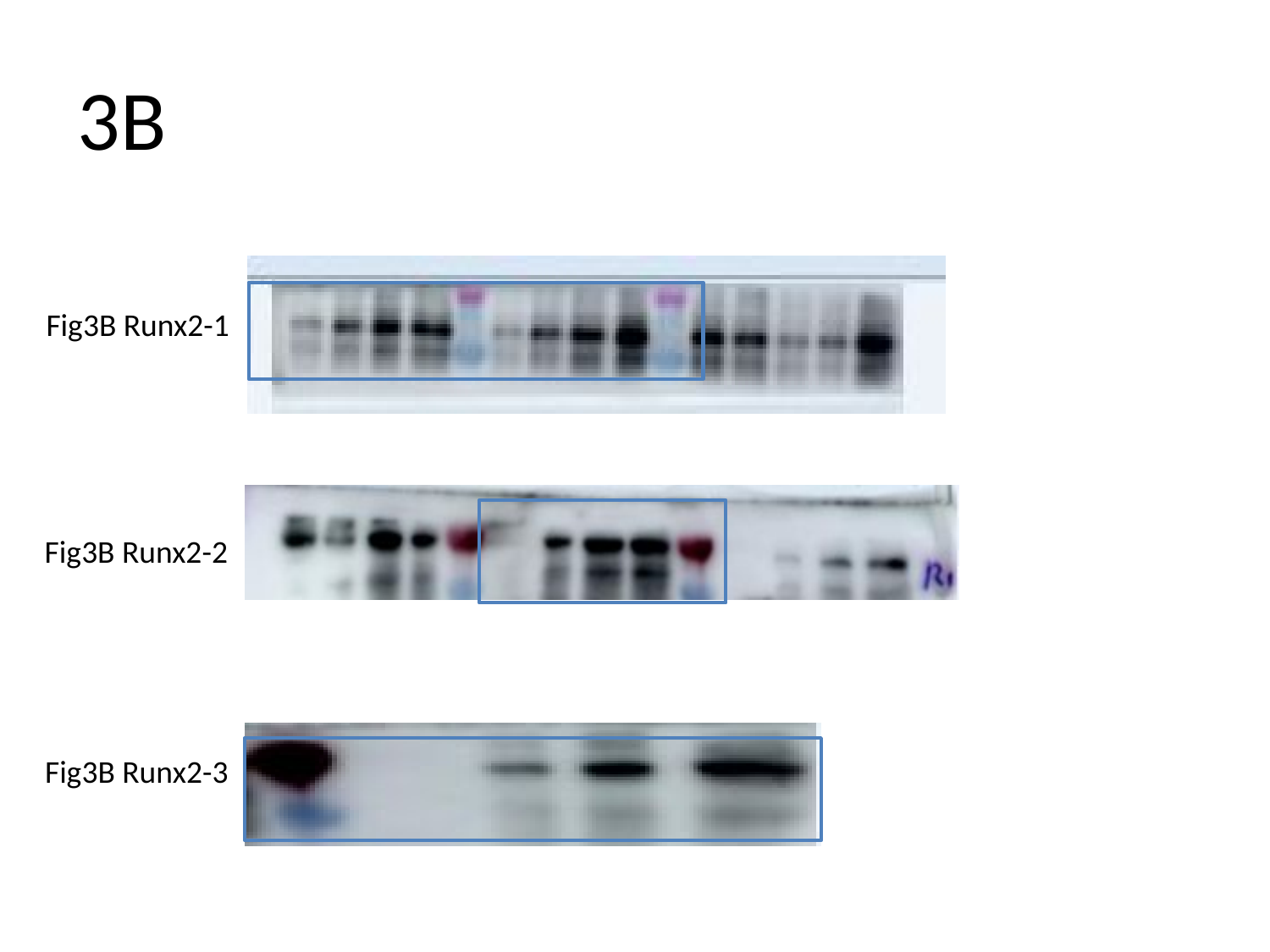

3B
Fig3B Runx2-1
Fig3B Runx2-2
Fig3B Runx2-3

## Slide 6
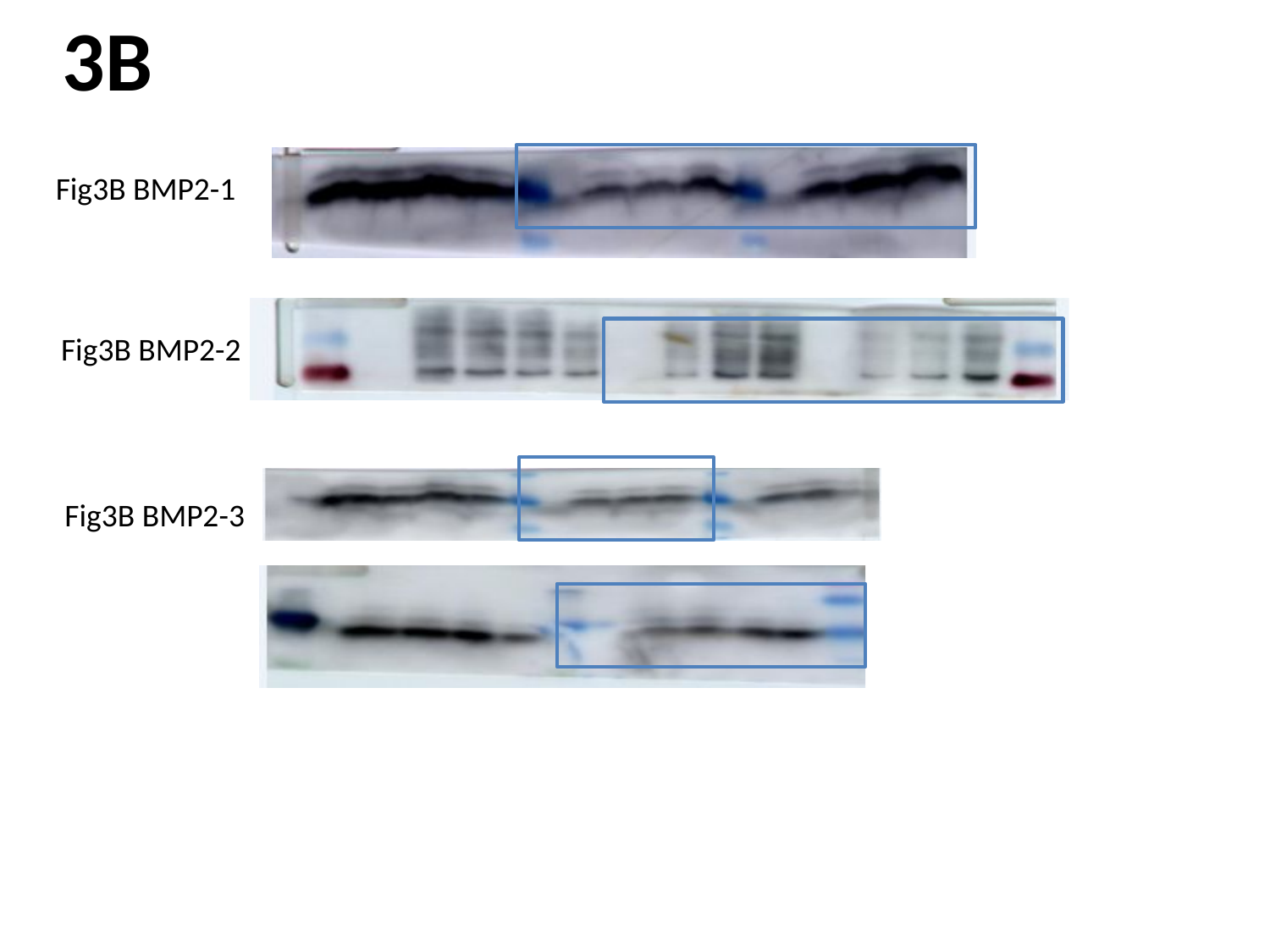

3B
Fig3B BMP2-1
Fig3B BMP2-2
Fig3B BMP2-3

## Slide 7
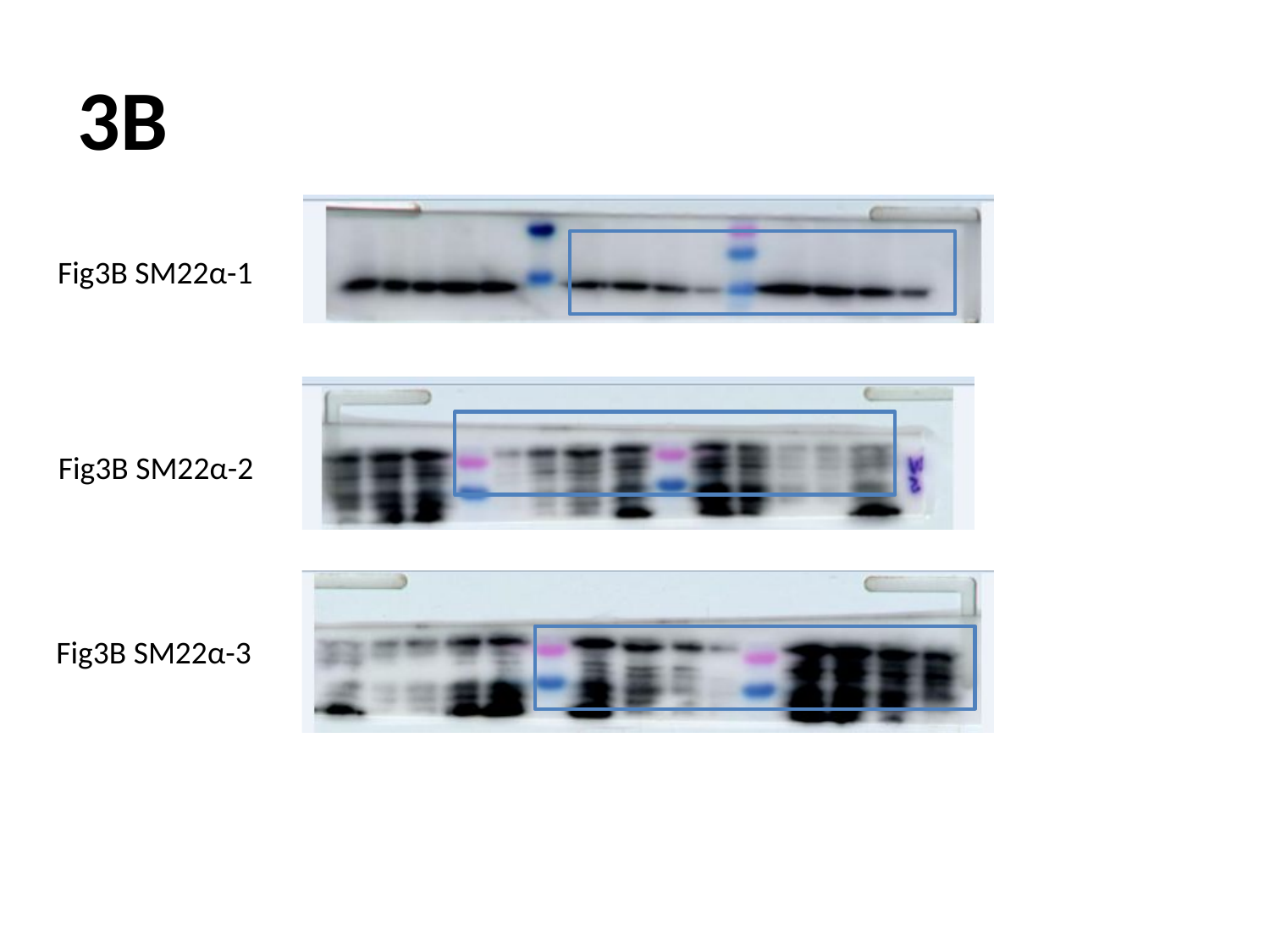

3B
Fig3B SM22α-1
Fig3B SM22α-2
Fig3B SM22α-3

## Slide 8
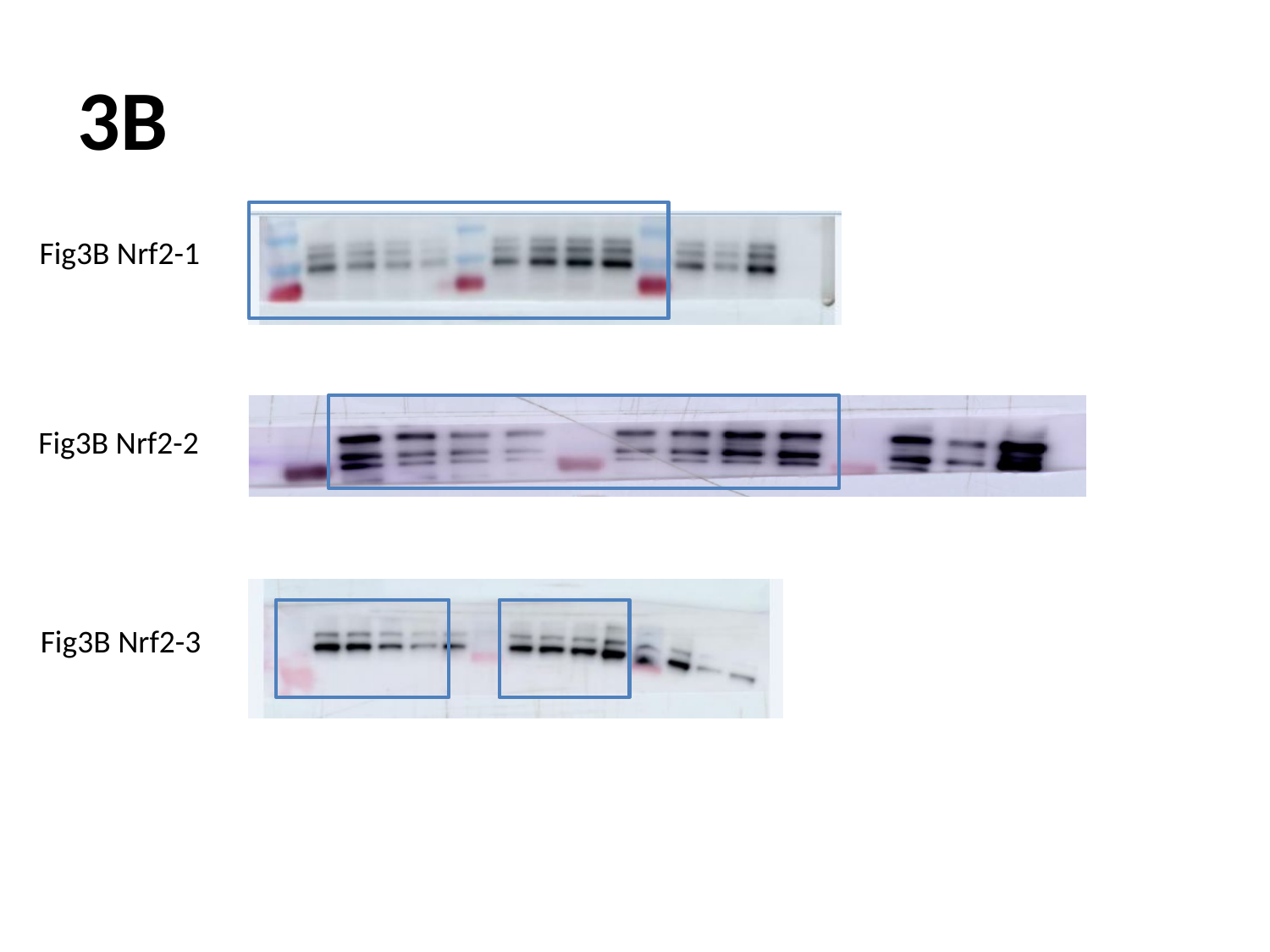

3B
Fig3B Nrf2-1
Fig3B Nrf2-2
Fig3B Nrf2-3

## Slide 9
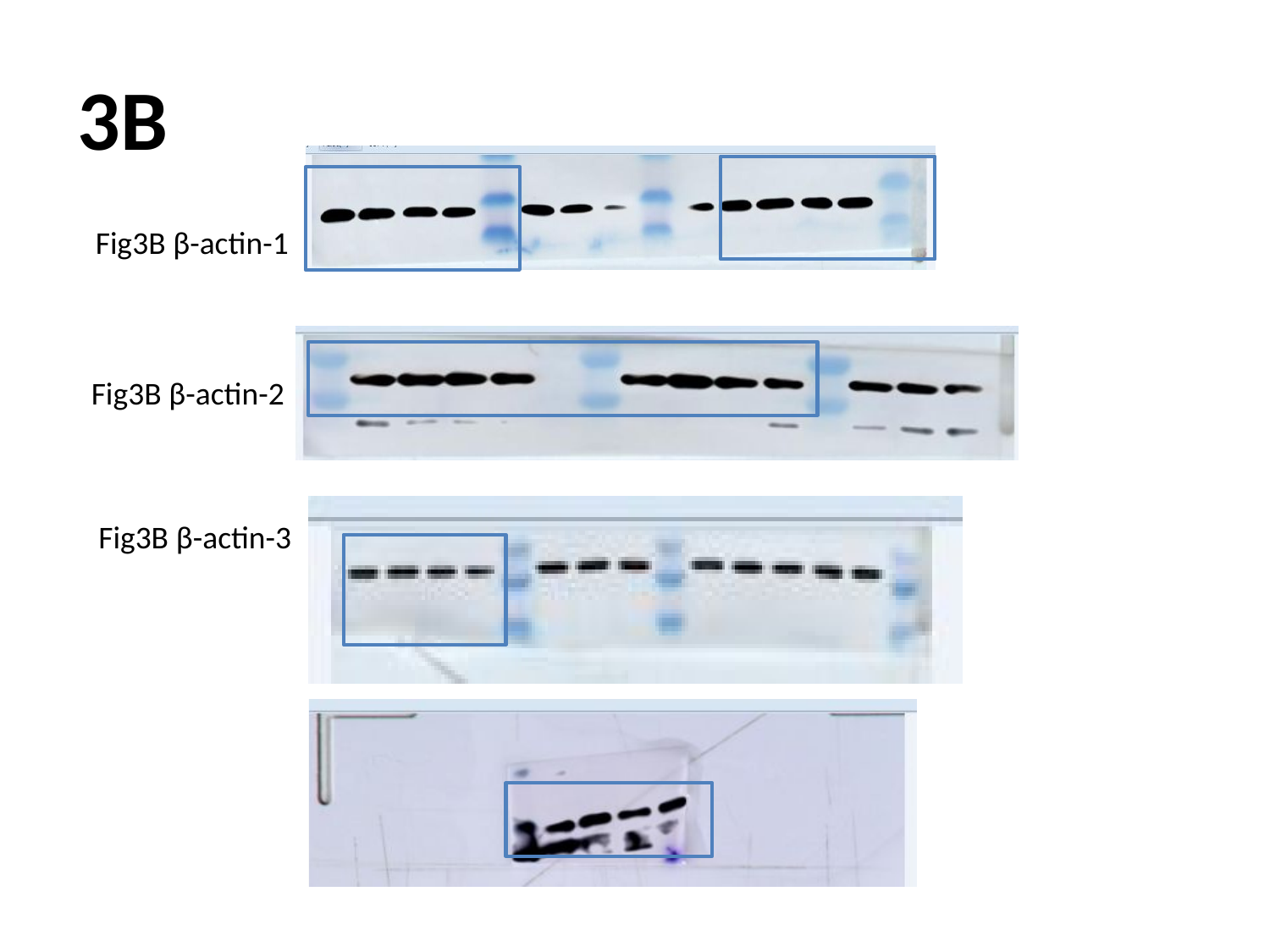

3B
Fig3B β-actin-1
Fig3B β-actin-2
Fig3B β-actin-3

## Slide 10
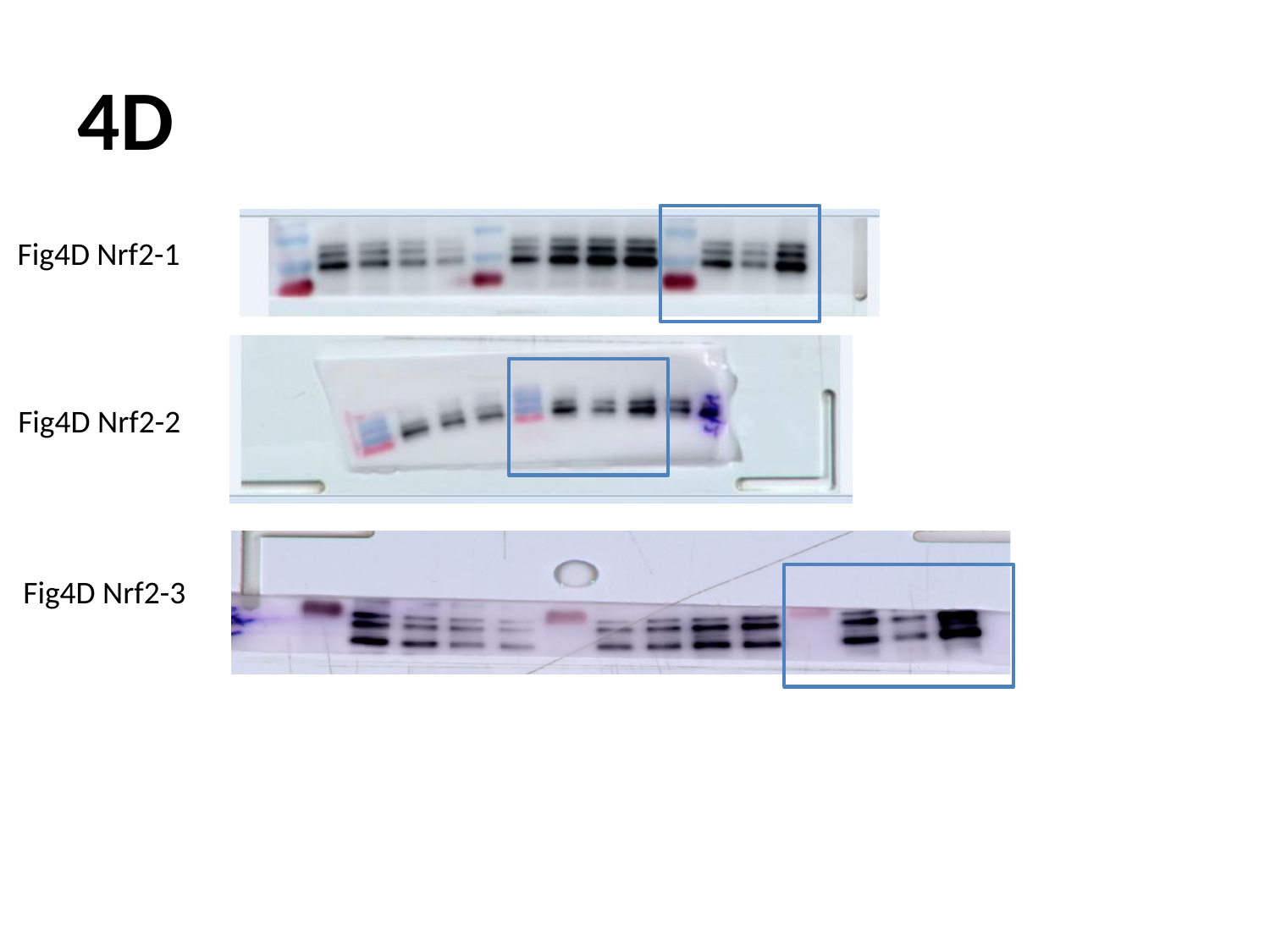

4D
Fig4D Nrf2-1
Fig4D Nrf2-2
Fig4D Nrf2-3

## Slide 11
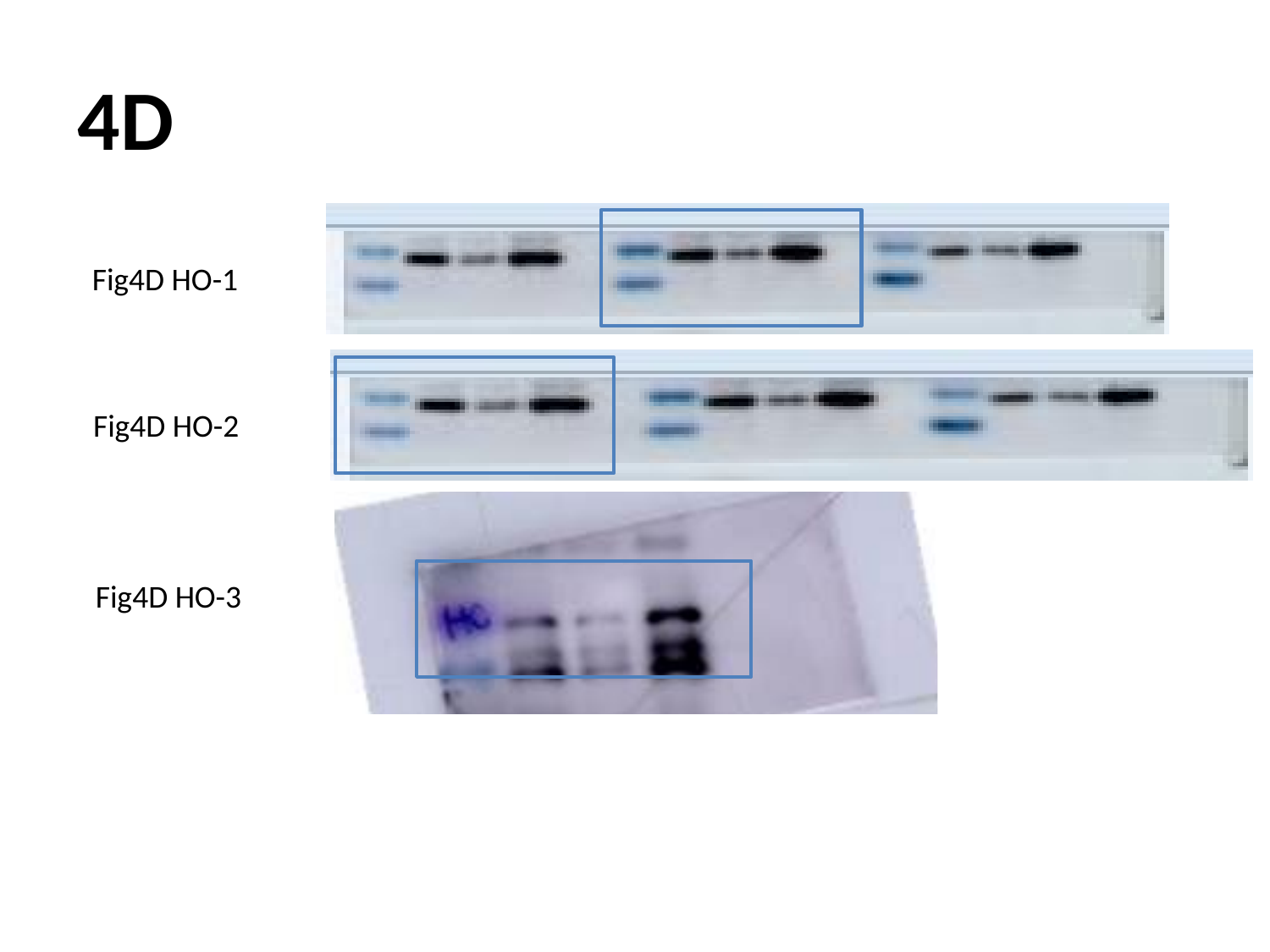

4D
Fig4D HO-1
Fig4D HO-2
Fig4D HO-3

## Slide 12
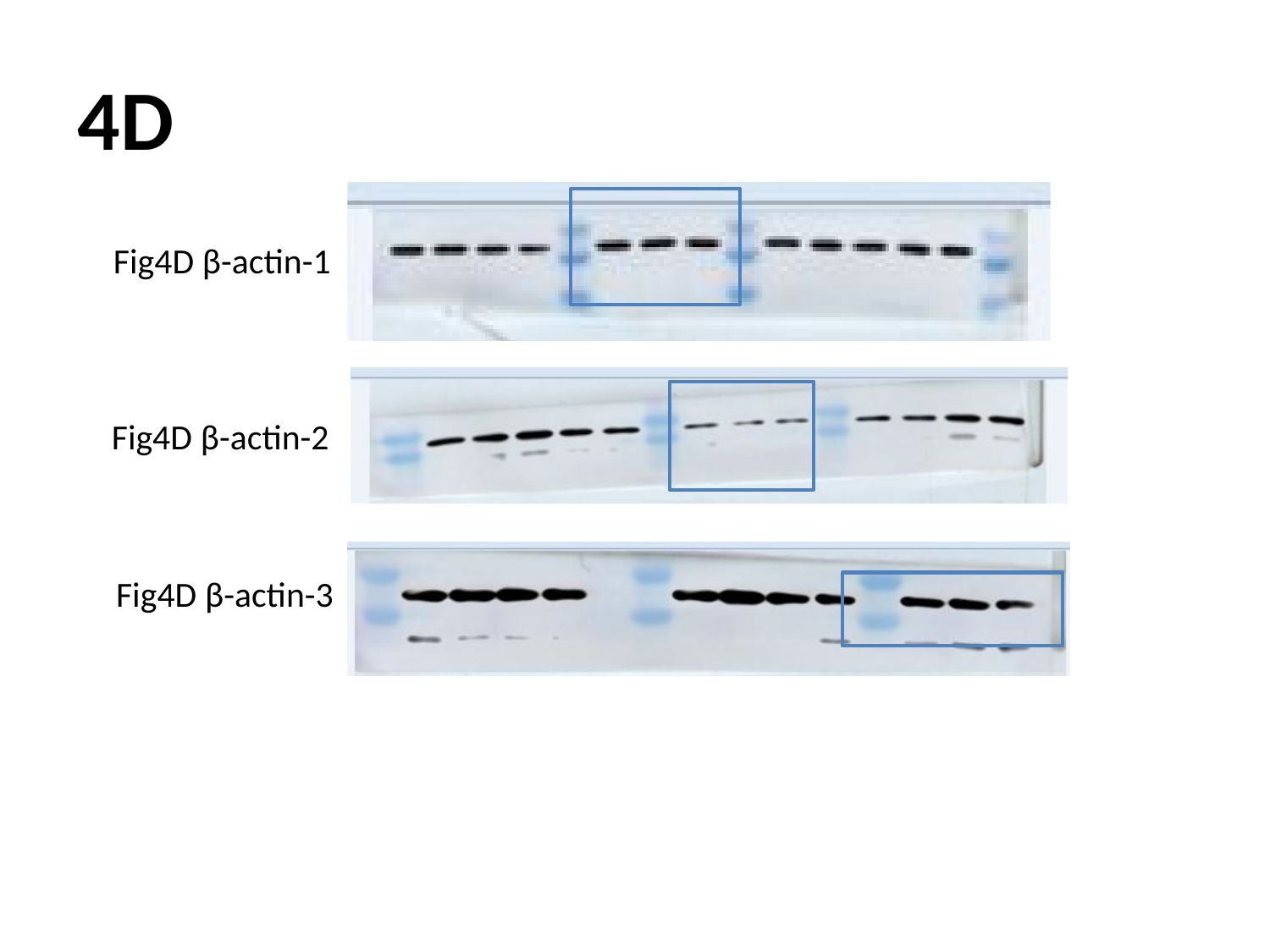

4D
Fig4D β-actin-1
Fig4D β-actin-2
Fig4D β-actin-3

## Slide 13
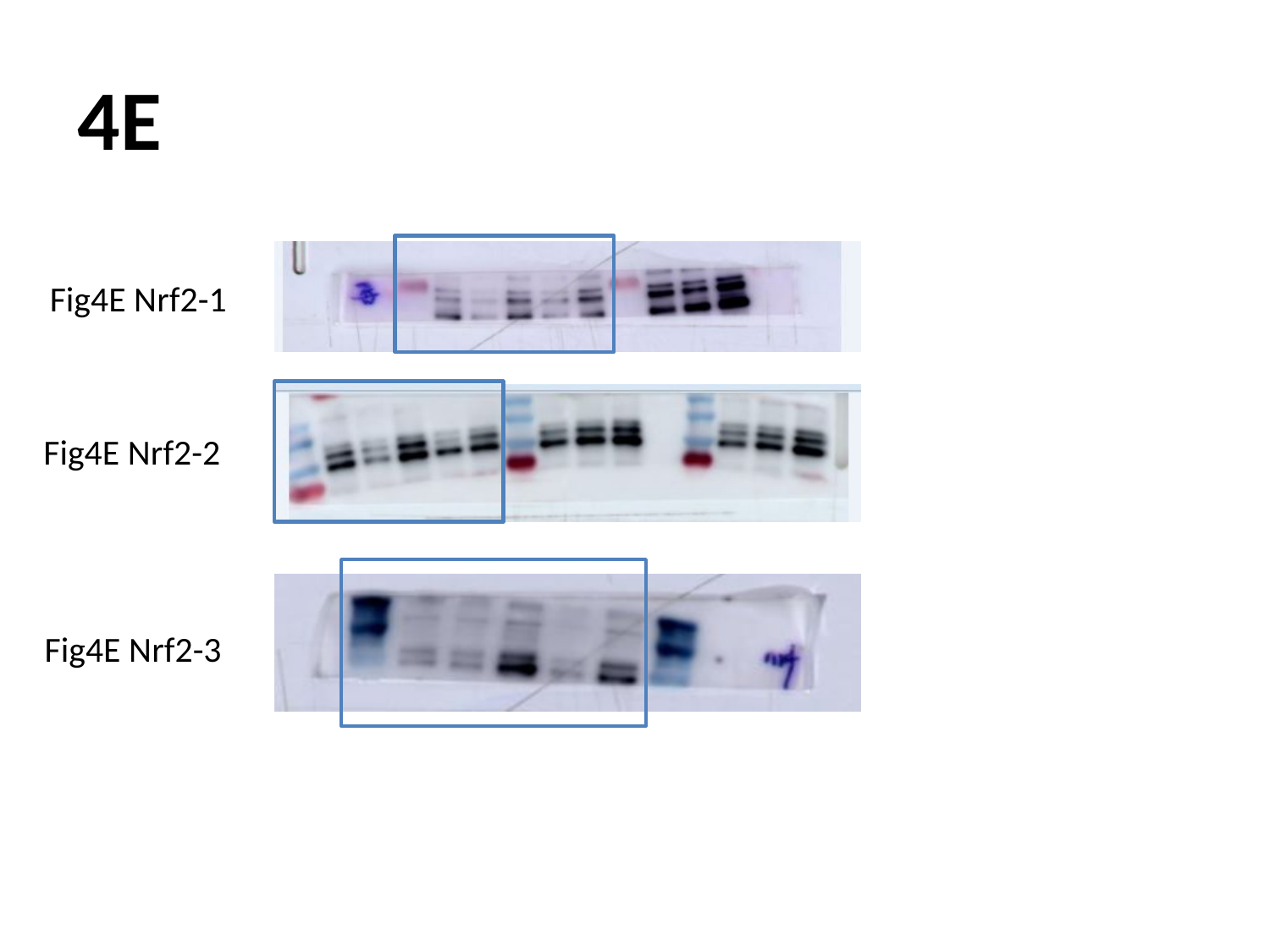

4E
Fig4E Nrf2-1
Fig4E Nrf2-2
Fig4E Nrf2-3

## Slide 14
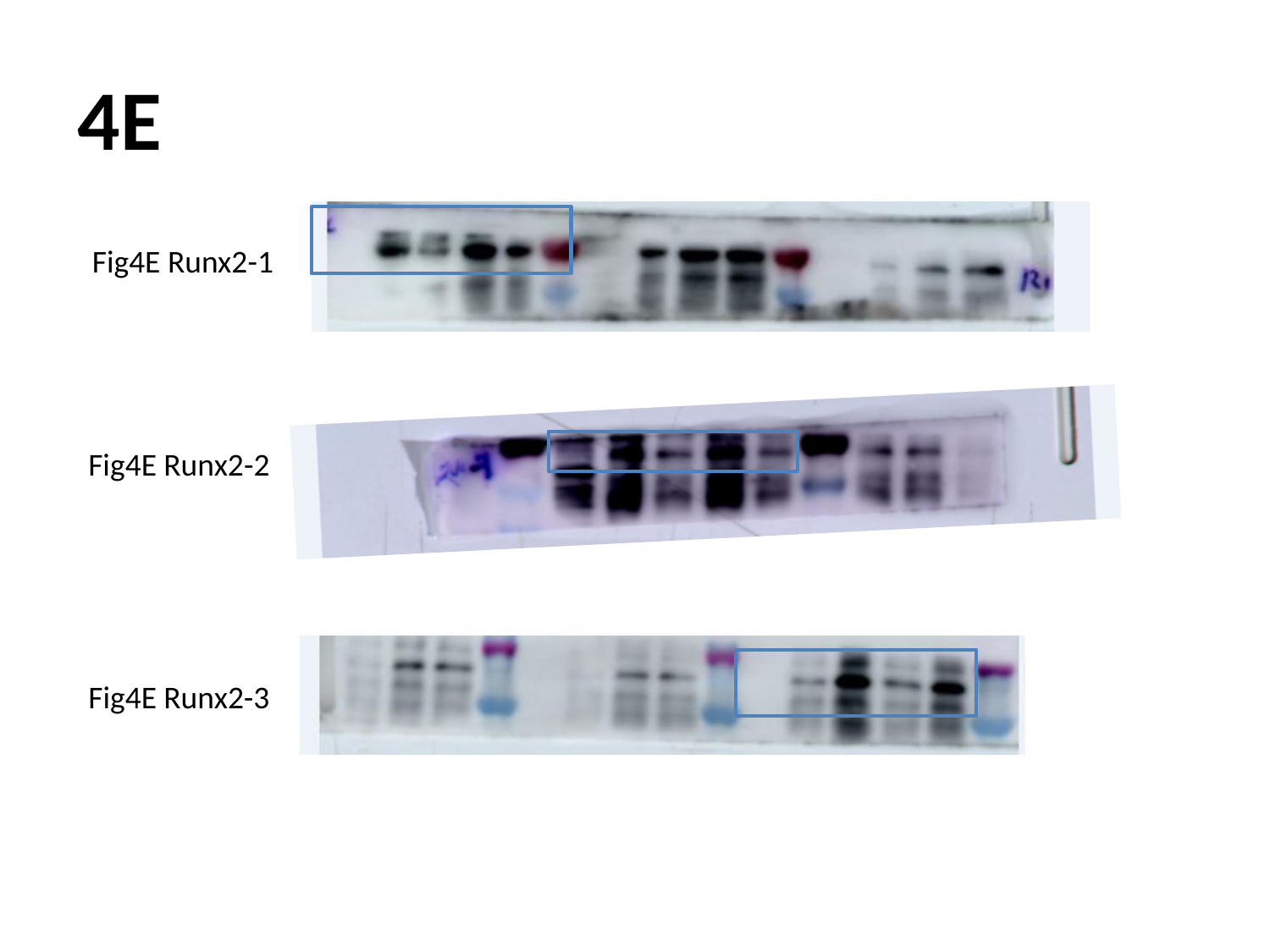

4E
Fig4E Runx2-1
Fig4E Runx2-2
Fig4E Runx2-3

## Slide 15
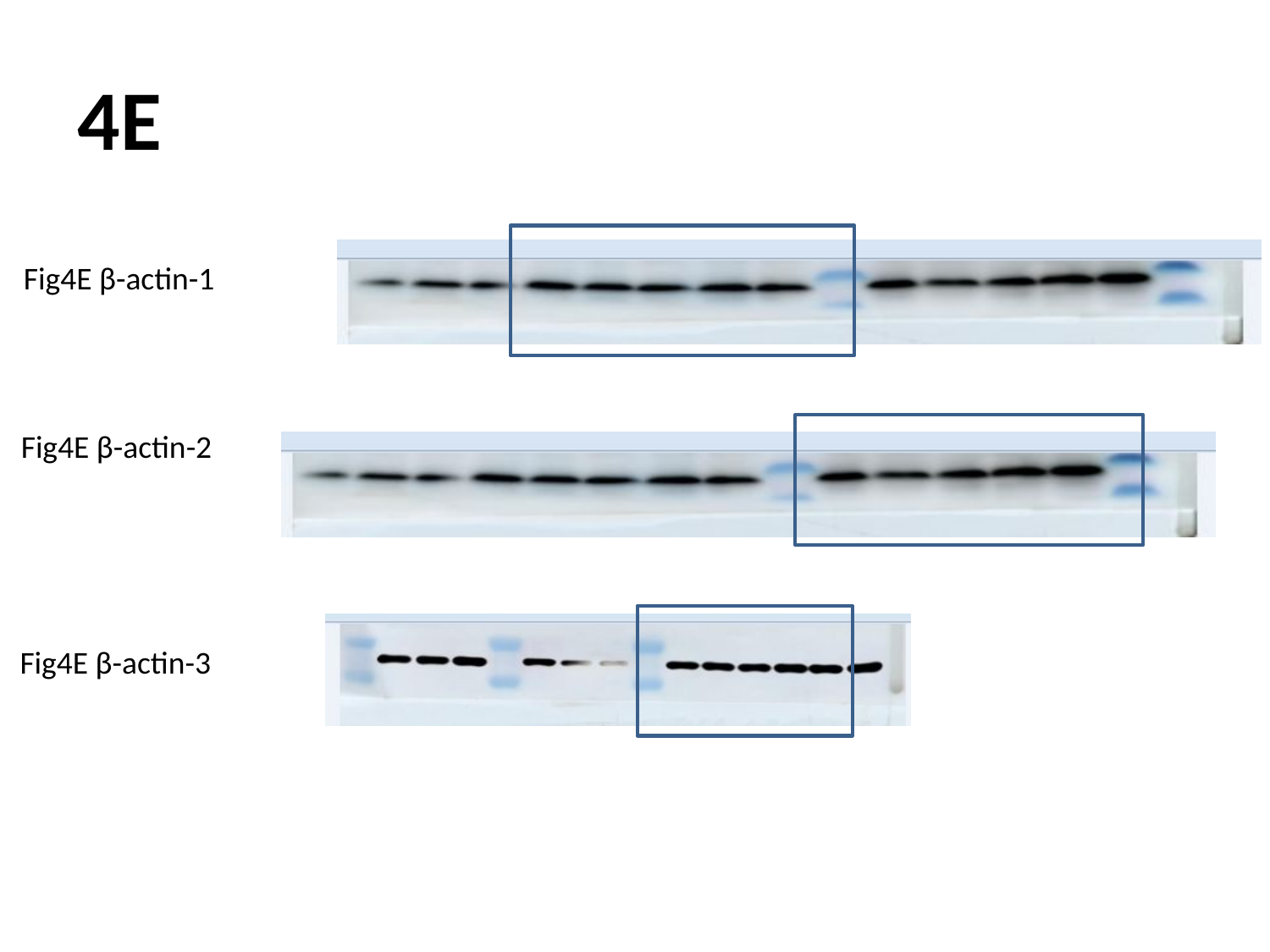

4E
Fig4E β-actin-1
Fig4E β-actin-2
Fig4E β-actin-3

## Slide 16
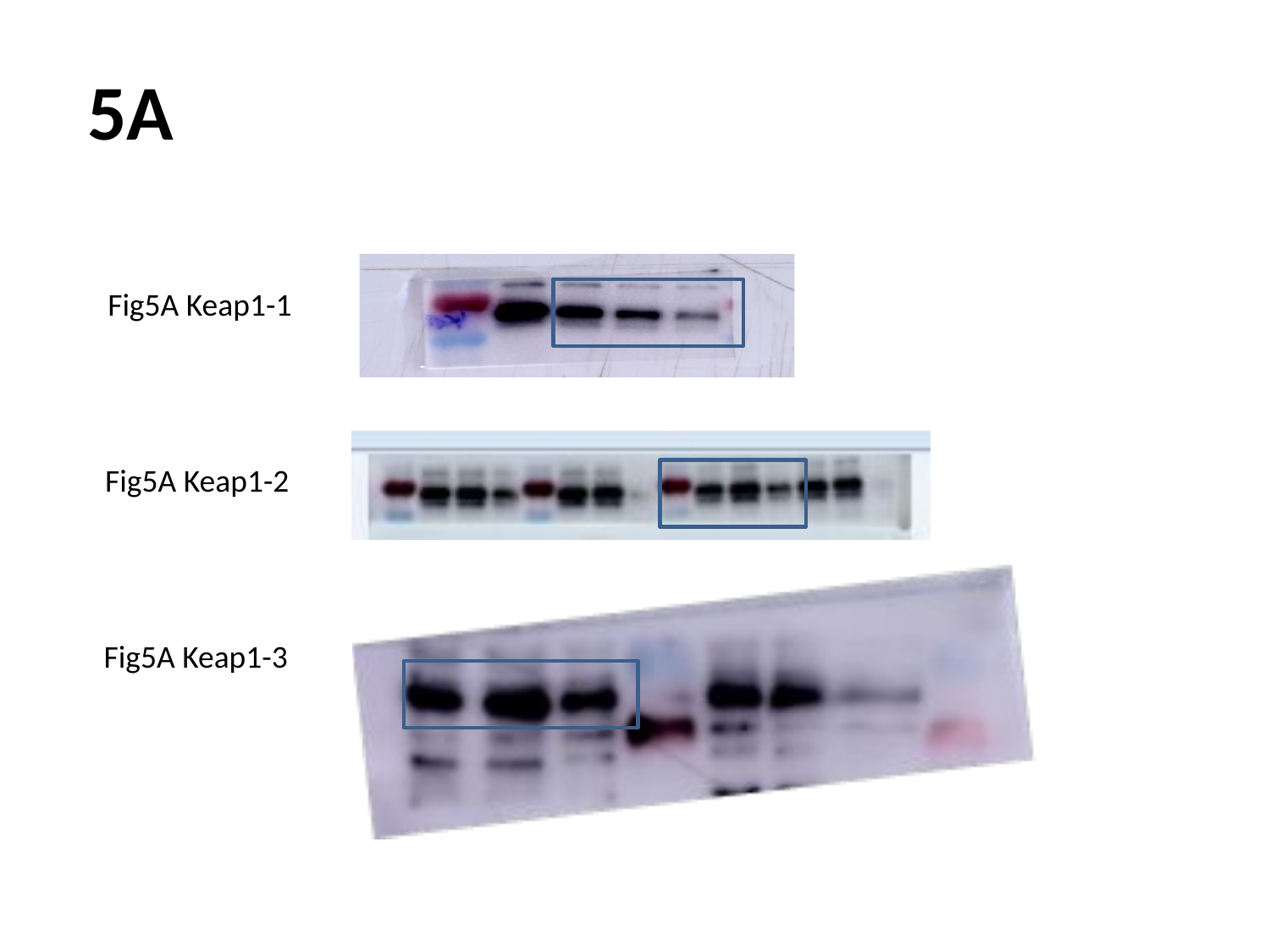

5A
Fig5A Keap1-1
Fig5A Keap1-2
Fig5A Keap1-3

## Slide 17
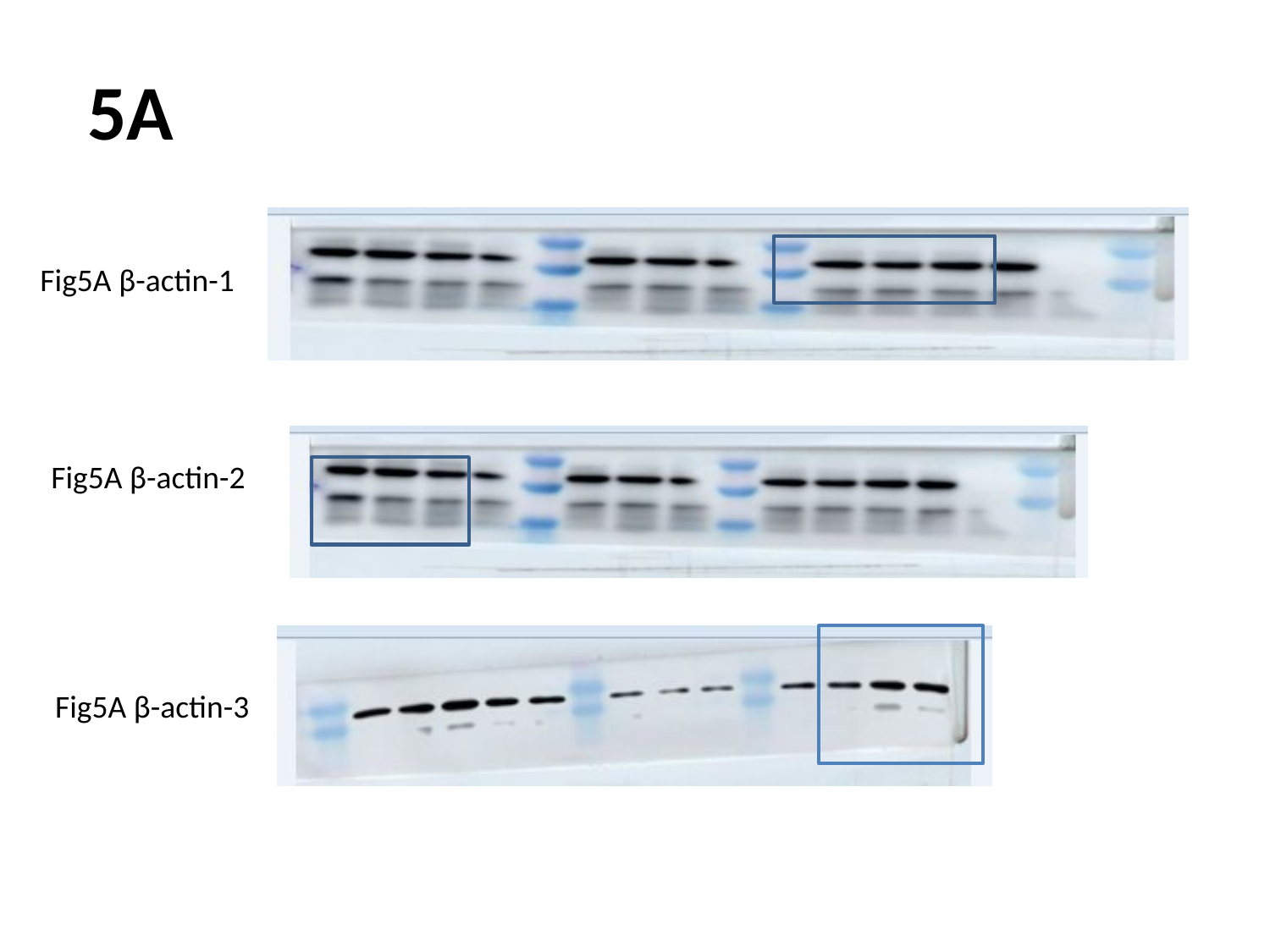

5A
Fig5A β-actin-1
Fig5A β-actin-2
Fig5A β-actin-3

## Slide 18
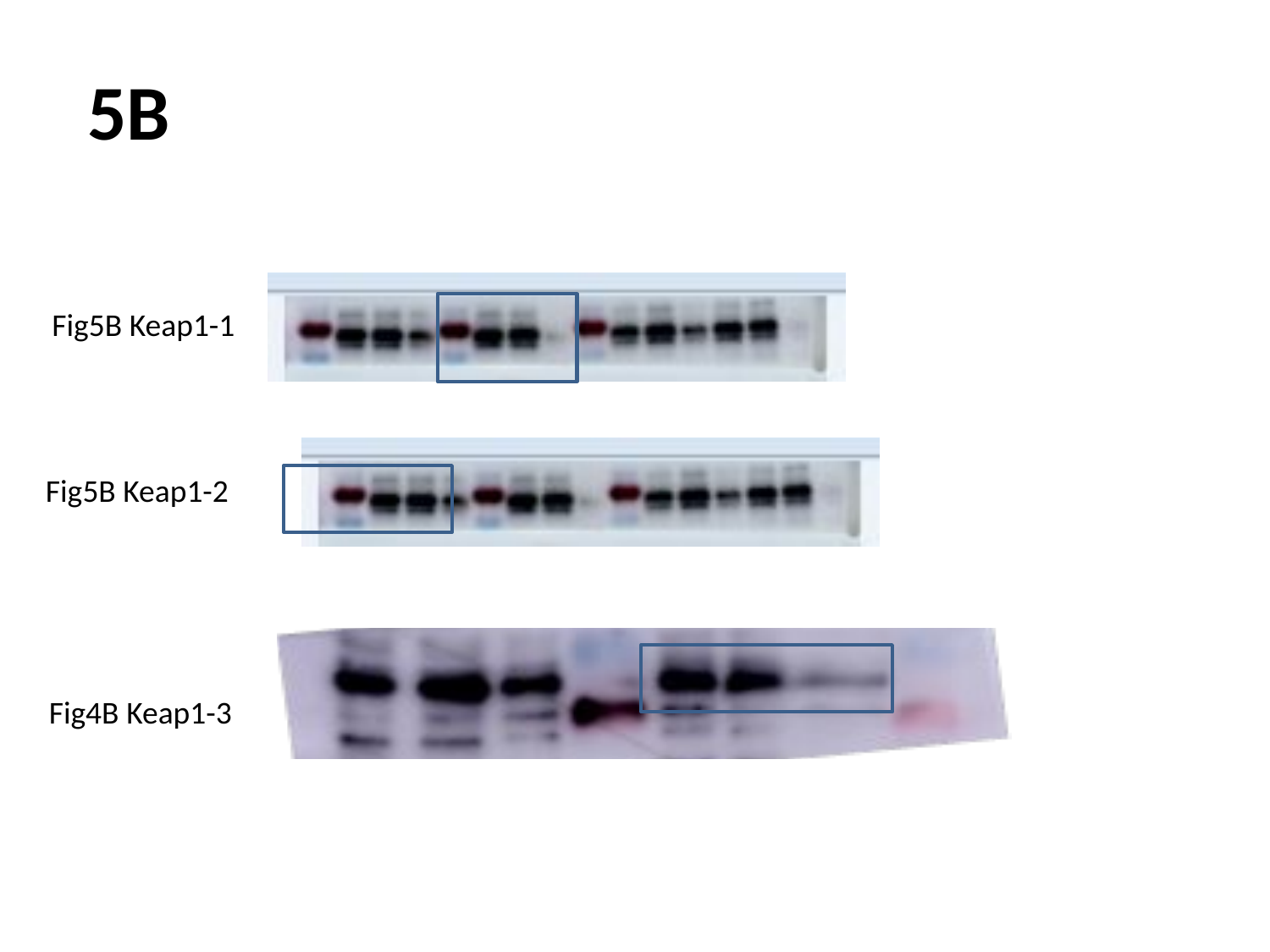

5B
Fig5B Keap1-1
Fig5B Keap1-2
Fig4B Keap1-3

## Slide 19
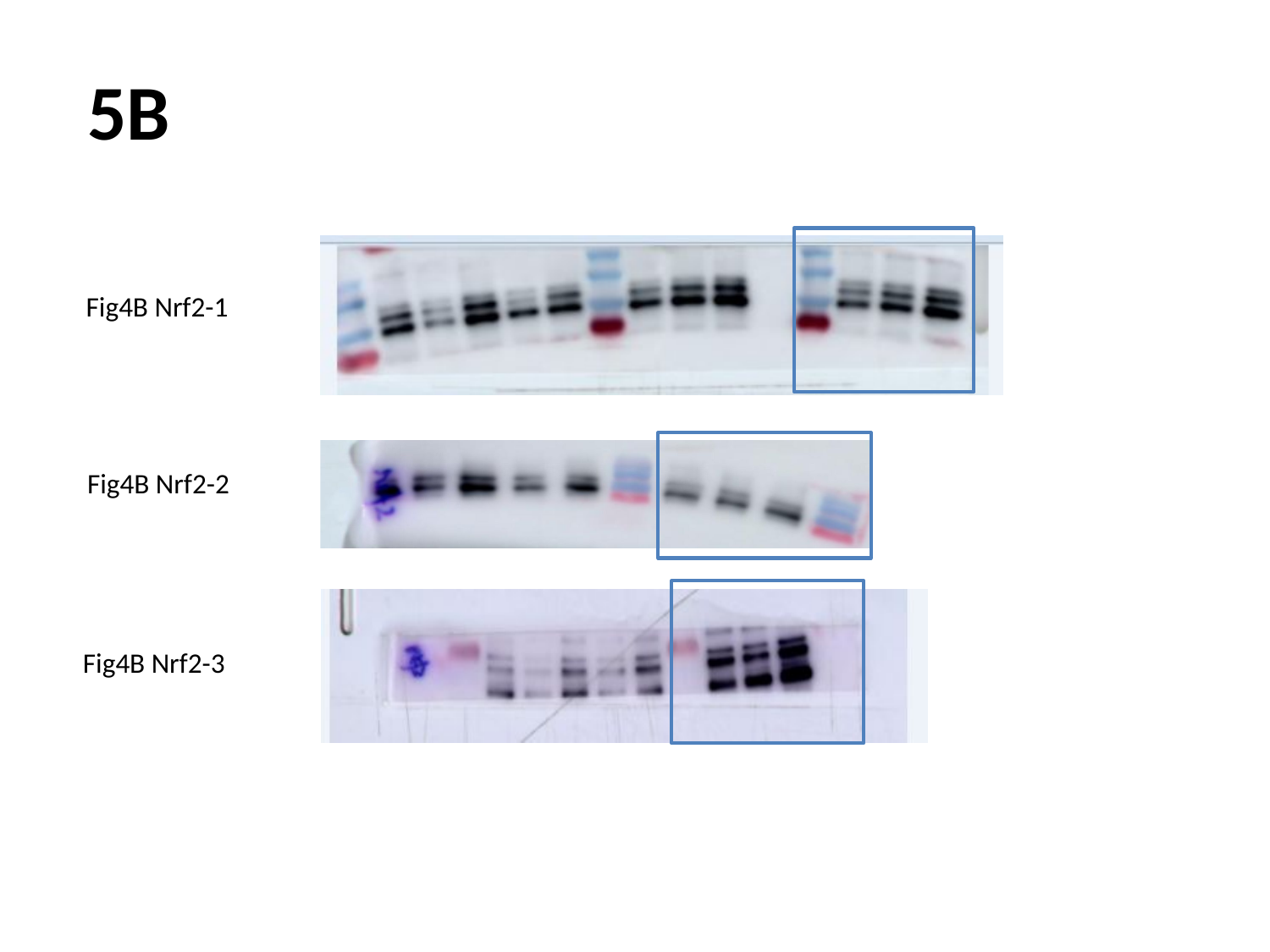

5B
Fig4B Nrf2-1
Fig4B Nrf2-2
Fig4B Nrf2-3

## Slide 20
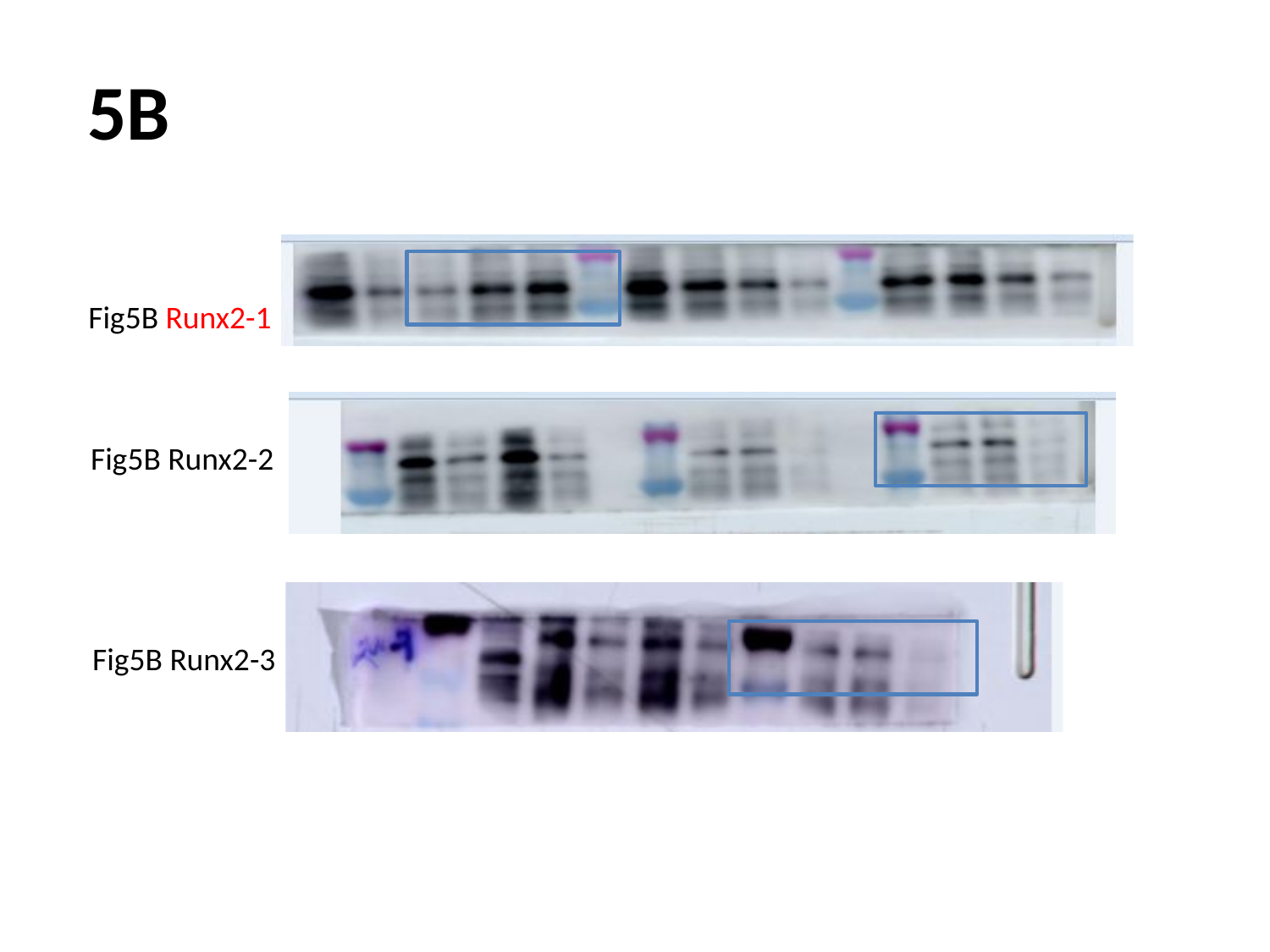

5B
Fig5B Runx2-1
Fig5B Runx2-2
Fig5B Runx2-3

## Slide 21
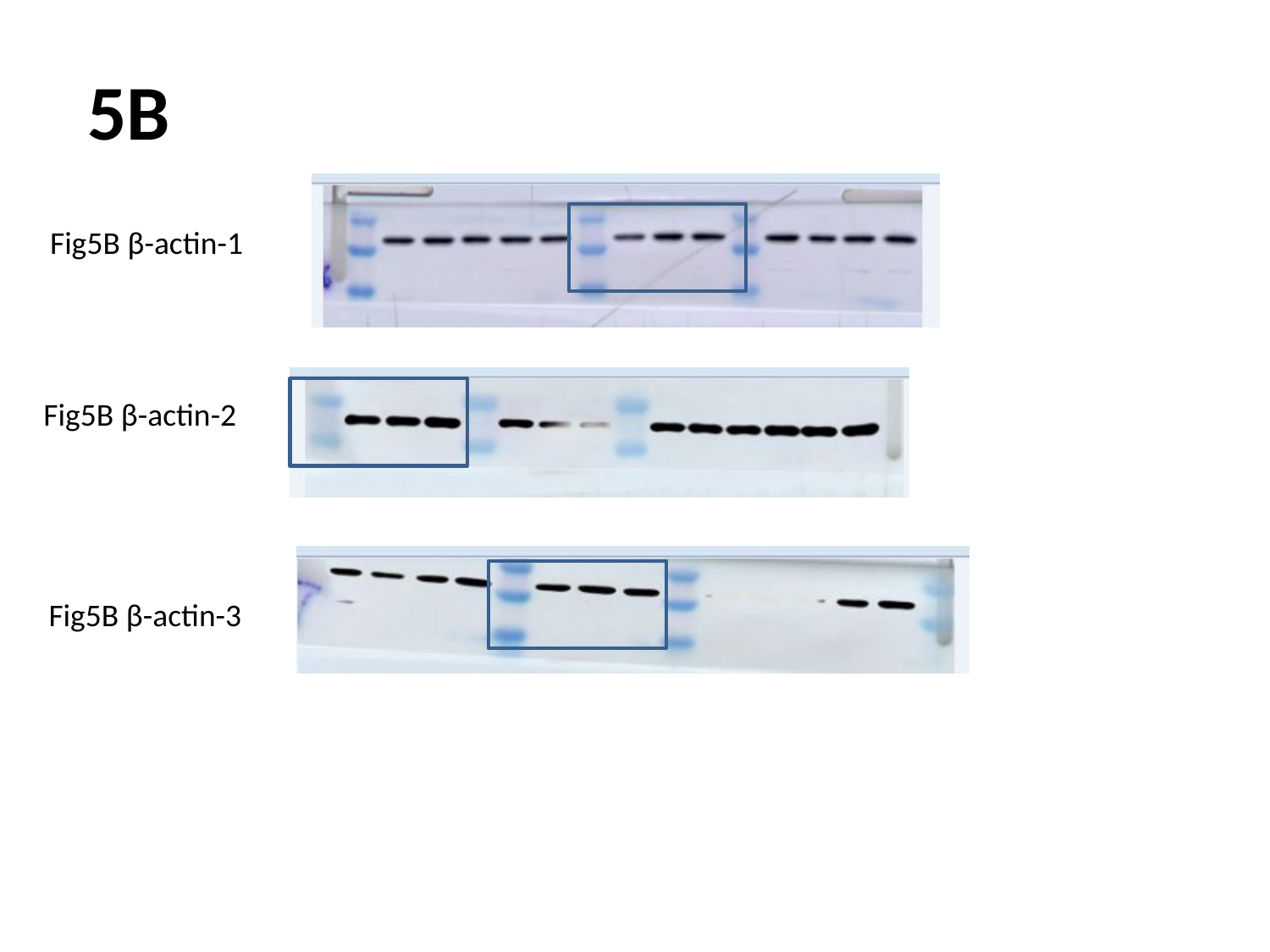

5B
Fig5B β-actin-1
Fig5B β-actin-2
Fig5B β-actin-3
